# Interplay of Energy and Charge Transfer in WSe_2_/CrSBr Heterostructures

**DOI:** 10.1021/acs.nanolett.5c03150

**Published:** 2025-08-22

**Authors:** José Roberto de Toledo, Caique Serati de Brito, Barbara L. T. Rosa, Alisson R. Cadore, César Ricardo Rabahi, Paulo E. Faria Junior, Ana Carolina Ferreira de Brito, Talieh S. Ghiasi, Josep Ingla-Aynés, Christian Schüller, Herre S. J. van der Zant, Stephan Reitzenstein, Ingrid D. Barcelos, Florian Dirnberger, Yara Galvão Gobato

**Affiliations:** † Physics Department, Federal University of São Carlos, 13565-905 São Carlos, SP Brazil; ‡ Institut für Festkörperphysik, Technische Universität, 10623 Berlin, Germany; ¶ Brazilian Nanotechnology National Laboratory (LNNano), 215006 Brazilian Center for Research in Energy and Materials (CNPEM), 13083-100 Campinas, SP Brazil; § Programa de Pós-Graduação em Física, Instituto de Física, Universidade Federal do Mato Grosso, 79070-900 Cuiabá, Brazil; ∥ Department of Physics, University of Central Florida, Orlando, Florida 32816, United States; ⊥ Department of Electrical and Computer Engineering, University of Central Florida, Orlando, Florida 32816, United States; # Brazilian Synchrotron Light Laboratory (LNLS), 215006 Brazilian Center for Research in Energy and Materials (CNPEM), 13083-100 Campinas, SP Brazil; @ Kavli Institute of Nanoscience, Delft University of Technology, 2628 CJ Delft, The Netherlands; △ Institut für Experimentelle und Angewandte Physik, Universität Regensburg, D-93040 Regensburg, Germany; ∇ Department of Physics, TUM School of Natural Sciences, 9184 Technical University of Munich, 85748 Garching, Germany; ° Zentrum für Quantum Engineering (ZQE), 9184 Technical University of Munich, 85748 Garching, Germany; • Munich Center for Quantum Science and Technology (MCQST), 9184 Technical University of Munich, 85748 Garching, Germany

**Keywords:** Two-Dimensional Magnets, CrSBr, Transition-Metal Dichalcogenides, Resonant Energy Transfer, Localized Excitons, Magneto-optics

## Abstract

van der Waals heterostructures (vdWHs) composed of transition-metal dichalcogenides (TMDs) and layered magnetic semiconductors offer great opportunities to manipulate the exciton and valley properties of TMDs. Here, we present magneto-photoluminescence (PL) studies in a WSe_2_ monolayer (ML) on a CrSBr crystal, an anisotropic layered antiferromagnetic semiconductor. Our results reveal the unique behavior of each of the ML-WSe_2_ PL peaks under a magnetic field that is distinct from the pristine case. An intriguing feature is the clear enhancement of the PL intensity that we observe each time the external magnetic field tunes the energy of an exciton in CrSBr into resonance with one of the optical states of WSe_2_. This result suggests a magnetic field-controlled resonant energy transfer (RET) beyond other effects reported in similar structures. Our work provides deep insight into the importance of different mechanisms in magnetic vdWHs and underscores its great potential for light harvesting and emission enhancement of two-dimensional materials.

## Graphical abstract

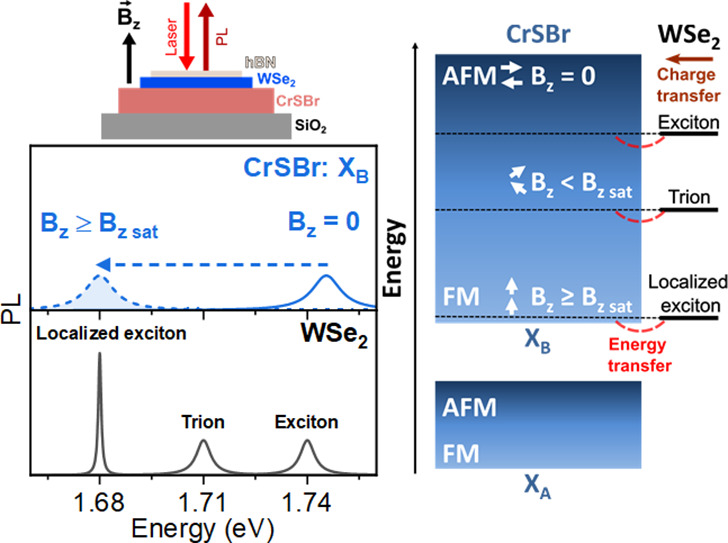

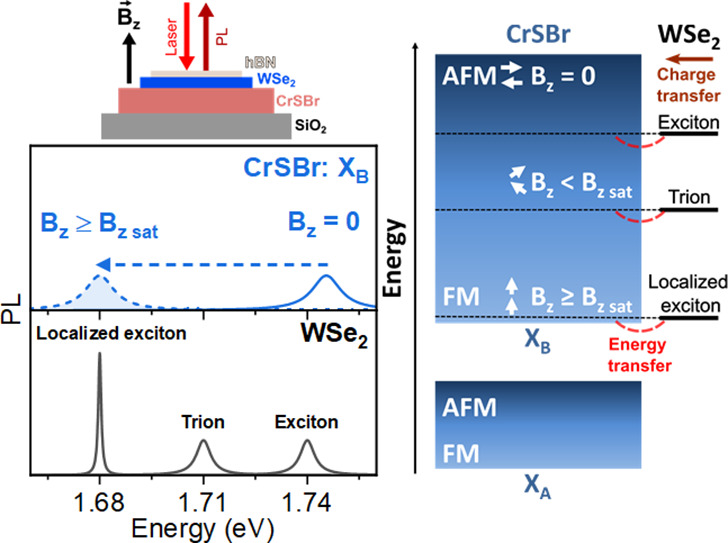

Two-dimensional (2D) magnetic materials have attracted great attention in the last few years, offering a new platform for studying fundamental properties of magnetism in low dimensions and for possible applications in spintronics.^1–8^ Among those, CrSBr has received increasing attention in the past few years because of its inherently coupled magnetic and optical properties.^6–9^ CrSBr is an air-stable quasi-1D van der Waals (vdW) semiconductor material with a direct band gap of about 1.5 eV.^6,10,11^ It has an orthorhombic crystal structure with a rectangular unit cell in the â−b̂ plane stacked along the ĉ direction.^6^ The CrSBr monolayer (ML) is ferromagnetic (FM).^6^ The interlayer exchange coupling between the layers favors an A-type antiferromagnetic order (AFM) with a Néel temperature of approximately 135 K.^7,12–20^ Its electronic band structure and consequently the energy of excitons are both very sensitive to the interlayer magnetic exchange interaction which allows probing its magnetic order by magneto-optical spectroscopy.^9,21^ CrSBr has two anisotropic emissions related to the fundamental bright exciton (labeled A exciton) and to a higher-energy exciton (labeled B exciton) at around 1.36 and 1.76 eV, respectively.^7,9,11,20–22^ The A exciton is tightly bound while the B exciton has a decreased spatial localization.^11^ Furthermore, both excitons show a red shift in their PL peak energy with increasing magnetic field up to a field-induced FM state.^7,9,21–24^

Very interesting material design opportunities appear when 2D magnetic materials can also be combined with nonmagnetic materials such as ML-TMD to modify their exciton and valley properties using magnetic exchange interaction and charge transfer effects.^8,25–30^ Several previous studies were performed in different vdWH’s composed of 2D FM materials with perpendicular magnetization such as CrBr_3_, CrI_3_, and ML-TMDs and have revealed important changes in the properties of ML-TMDs such as valley splitting and the degree of polarization under zero magnetic field.^27–31^ Recent studies were focused on ML-MoSe_2_/CrSBr, a type-III vdWH, and have revealed important modifications in their physical properties, which were associated with magnetic proximity and charge transfer effects.^21,32^

There is also increasing interest in 2D materials such as ML-WSe_2_ to generate single-photon emitters (SPEs) for possible applications in photonic quantum technologies.^33–37^ In particular, several 2D magnetic materials (Cr_2_Ge_2_Te_6_, CrI_3_, and NiPS_3_) have been used as substrates to modify the optical properties of SPEs, evidencing an enhancement of the *g*-factors and circular polarization, offering an exciting platform for designing quantum devices for implementing photonic quantum networks.^38–40^ In this context, the investigation of ML-WSe_2_/CrSBr could reveal new opportunities to modify the optical properties of localized excitons in WSe_2_ in advanced quantum light sources.

Here, we investigate the excitonic properties of ML-WSe_2_ on CrSBr using low-temperature magneto-PL techniques. Different contributions of the CrSBr layer are observed in the magnetic field dependence of PL peaks in the WSe_2_/CrSBr heterostructure. In addition to charge transfer effects, we observe clear PL signatures each time the B exciton (labeled X_*B*_) of CrSBr comes into resonance with an exciton state in the ML-WSe_2_. As the PL peak energies of CrSBr can be controlled by the applied magnetic field, the resonant condition of the energy states of CrSBr and ML-WSe_2_ can be tuned by this external parameter, resulting in changes in the PL properties of ML-WSe_2_/CrSBr. Remarkably, the PL of the sharp emission peaks in ML-WSe_2_/CrSBr shows significant intensity enhancement after the field-induced ferromagnetic state of CrSBr. Moreover, the PL intensity of excitons and trions in ML-WSe_2_ is also enhanced with different out-of-plane magnetic fields. We suggest that these results could be explained by the resonant energy transfer (RET) effect involving X_*B*_ in CrSBr. Our studies point out the importance of different mechanisms to manipulate optical properties of excitonic states of ML-TMDs in magnetic vdWHs.

Our sample consists of ML-WSe_2_ on bulk CrSBr, capped by a thin layer of hexagonal boron nitride (hBN). Figure 1(a) presents an optical microscope image of the sample showing the crystal orientations, â and b̂ , of the CrSBr crystal. Figure 1(b–d) presents the typical PL spectra for the pristine CrSBr and WSe_2_/CrSBr for a laser excitation energy of 1.88 eV at 3.6 K. Several emission peaks are observed below 1.4 eV and are associated with the A exciton (labeled X_*A*_) in CrSBr.^6,12,21^ Additionally, we also detect a much weaker emission band in the range of 1.60 to 1.77 eV (Figure 1(b)) which is attributed to the X_*B*_ exciton in CrSBr.

**Figure 1:**
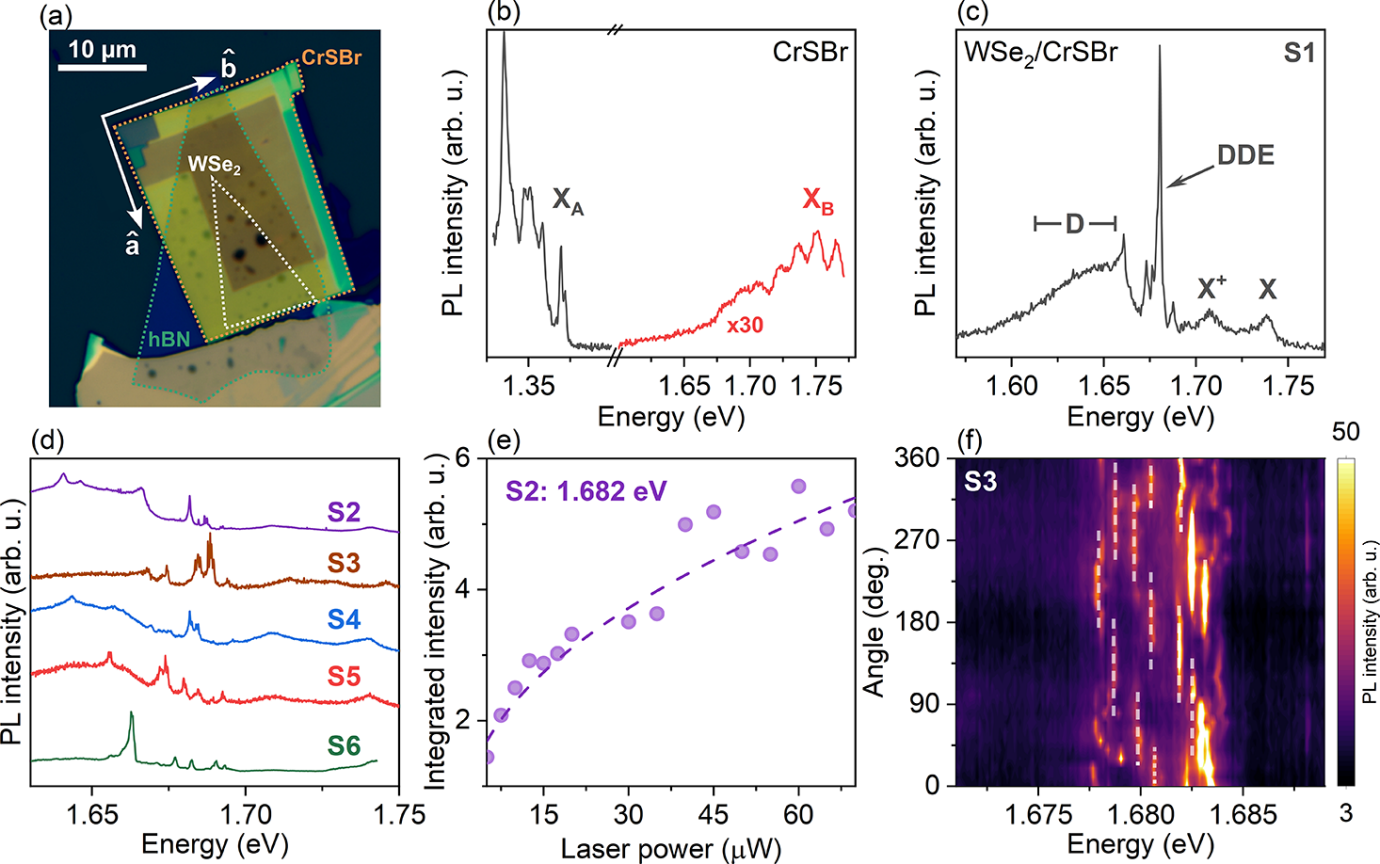
(a) Optical microscopy image of the ML-WSe_2_/CrSBr sample, indicating the orientation of the CrSBr crystallographic axes â and b̂ . (b) Typical PL spectrum of bulk CrSBr, showing the emission of the A (black curve) and B excitons (red curve). (c) PL spectrum of the WSe_2_/CrSBr heterostructure, showing several emission peaks from the WSe_2_ layer. (d) PL spectra for different laser positions, labeled S2–S6, showing several sharp PL peaks (labeled DDE). (e) Laser power dependence of the PL intensity for a sharp emission peak at 1.682 eV. (f) Color-coded map of the PL intensity of the DDE states as a function of the in-plane polarization angle, revealing several doublet peaks. All PL data were obtained using linearly polarized laser excitation along the b̂ axis with energy of 1.88 eV at 3.6 K.

Figure 1(c,d) shows the emission peaks from WSe_2_/CrSBr which are close to the spectral range of the emission of *X*_*B*_ in CrSBr. The bright exciton (X) and trion (X^+^) PL peaks of ML-WSe_2_ are observed at around 1.738 and 1.707 eV, respectively. Furthermore, we also observe a broad PL band that is associated with the emission of defects and labeled as a defect band (D) in panel (c). Several sharp emission peaks are also revealed below 1.68 eV for different sample positions as shown in Figure 1(d). As expected, the PL spectra change depending on laser position due to the presence of different local strain.^8,33–36,41–43^ They are associated with defect dark exciton states (DDE)^34^ which are promising candidates for single-photon emitters (SPEs). Figure 1(e) shows the integrated PL intensity of one of the sharp PL peaks as a function of the laser power. A saturation trend is observed which clearly confirms the localized nature of these emission peaks.^33,34,36,44–47^ Figure 1(f) shows a typical color-coded map of the linearly polarized emission intensity of the DDE peaks as a function of the angle of in-plane polarization. We evidence several doublet emission peaks with orthogonal linear polarization, showing a typical zero-field splitting of δ ≈ 0.65 meV (details in equation (S1) in the SI file) similar to previous reports in the literature.^33,41,48–55^

In order to investigate the impact of the CrSBr layer on the exciton and valley properties of ML-WSe_2_, we have performed circular polarization-resolved PL measurements under an out-of-plane magnetic field (B_*z*_). Figure 2(a,b) shows the color-coded map of the circular polarization-resolved PL intensity of the pristine CrSBr as a function of B_*z*_, using linearly polarized laser excitation parallel to the b̂ axis of CrSBr at 3.6 K. The CrSBr PL peak energy of X_*A*_ shows a red shift of about 16 meV (Figure S9 in the SI) after the magnetic field-induced phase transition of CrSBr with a saturation field of |*B*_*z sat*_| ≈ 2.2 T. On the other hand, the X_*B*_ emission (Figure 2(b)) features a higher-energy red shift of about 80 meV (Figure S9) with increasing magnetic field, which is similar to very recent reports in the literature.^22–24^ Figure 2(c) presents a schematic view of the type-III band alignment of the heterostructure to illustrate the charge transfer effect, which can affect the PL intensity of WSe_2_/CrSBr.

**Figure 2:**
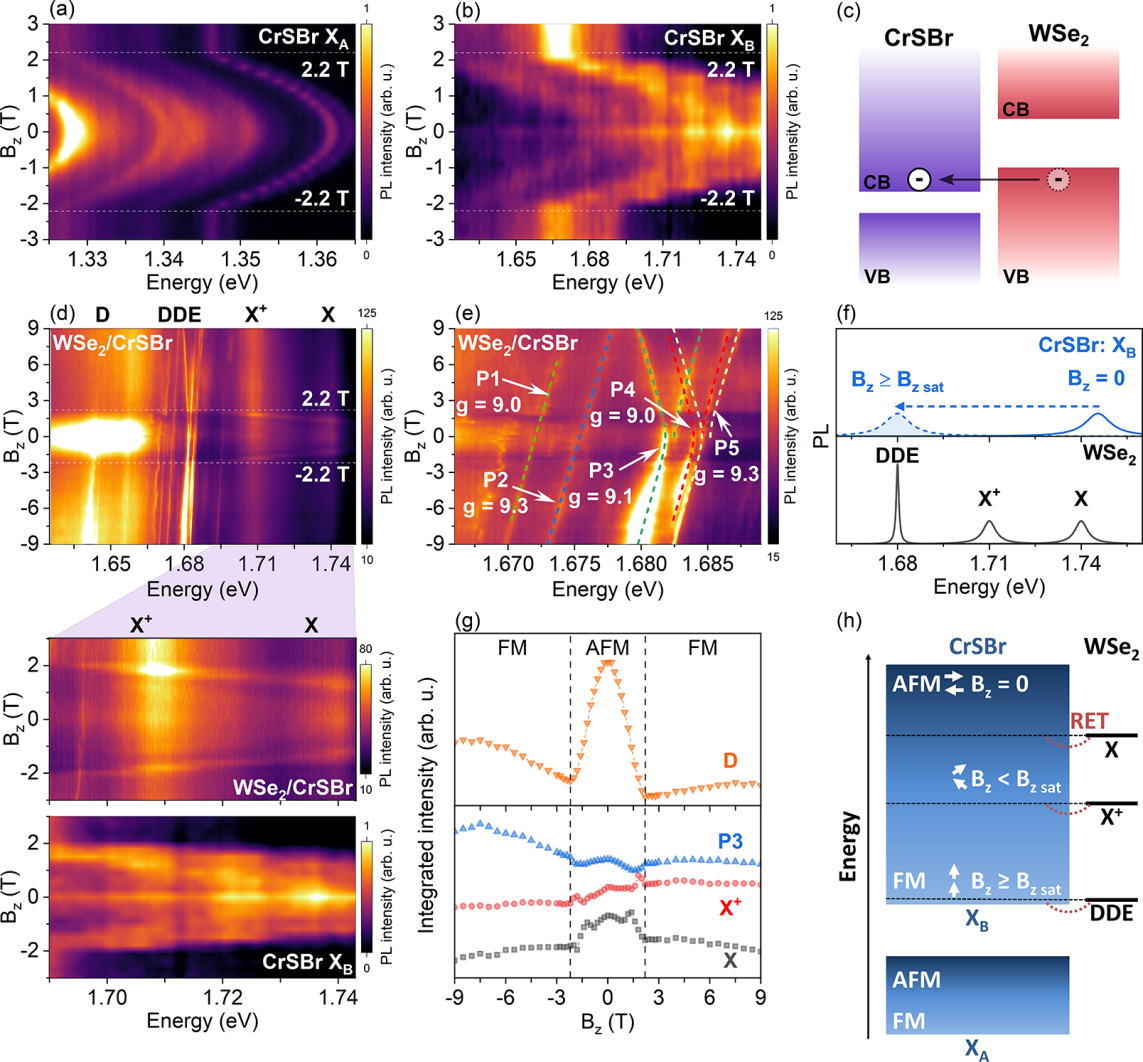
(a,b) Color-coded map of the circularly polarized PL intensity for the fundamental (X_*A*_) and X_*B*_ in pristine CrSBr flakes as a function of a magnetic field applied along the ĉ axis (labeled B_*z*_). (c) Schematic representation of the type-III band alignment for the WSe_2_/CrSBr heterostructure showing the charge transfer effect between the layers. (d) Color-coded map of the circularly polarized PL spectra in the WSe_2_/CrSBr heterostructure as a function of the magnetic field. Magnified view of the color-coded map showing anomalous changes in the PL intensity for ML WSe_2_ due to the resonant condition of excitonic states in ML WSe_2_ and X_*B*_ in CrSBr. (e) Enlarged view of the color-coded map in the spectral range corresponding to the emission of the localized excitons in panel (d). The dashed lines indicate the magnetic field dependence of the PL peak energies as a function of the magnetic field. (f) Schematic representation of the pristine CrSBr and ML-WSe_2_ PL bands, showing that by applying a magnetic field, the emission of the X_*B*_ can be tuned into resonance with the emissions of WSe_2_ in the heterostructure. (g) Magnetic field dependence of the integrated PL intensity for the D band, X, X^+^, and one of the DDE emission peaks (P3 emission on panel (e)). The vertical dashed lines indicate the saturation magnetic fields of CrSBr (|*B*_*z sat*_| ≈ 2.2 T). (h) Schematic drawing of the possible RET effect tuned by the magnetic field in the WSe_2_/CrSBr heterostructure. The PL data were obtained using a linearly polarized laser along the b̂ axis with energy of 1.88 eV. The σ^–^ PL component was collected for the positive magnetic field at 3.6 K.

Figure 2(d) shows a color-coded map of the circular polarization-resolved PL intensity of the WSe_2_/CrSBr heterostructure as a function of B_*z*_. We observe different modifications in the PL intensity for the emission peaks in ML-WSe_2_/CrSBr as compared to previous magneto-PL studies in MoSe_2_/CrSBr.^21^ In this previous study, a reduction of PL intensity of the MoSe_2_ trion/exciton peak accompanied by a change in relative intensity of the trion/exciton was observed after the magnetic phase transition of CrSBr.^21^ On the other hand, for the WSe_2_/CrSBr heterostructure, a clear enhancement of the PL intensity of the WSe_2_ X and X^+^ PL peaks is observed for magnetic fields that tune X_*B*_ in CrSBr into resonance with these peaks in WSe_2_ (for B_*z*_ < B_*z sat*_), suggesting a possible contribution of the RET effect. These effects are shown in more detail in the enlarged views of the color-coded map for the PL intensity, evidencing a clear correlation between changes in the PL intensity of WSe_2_ with the X_*B*_ PL peak energy. For B_*z*_ > B_*z sat*_, the changes in the PL intensity are dominated by the valley Zeeman effect and thermalization of carriers in the K and K′ valleys.^8,56–59^ Furthermore, a significant enhancement of the DDE PL peaks is clearly revealed at B_*z sat*_. On the contrary, the PL intensity of the D band, which is in the spectral range that cannot be tuned with X_*B*_, is reduced after the phase transition in CrSBr, and this effect is also explained by changes in the degree of charge transfer.^21^ Figure 2(g) shows the magnetic field dependence of integrated PL intensity for D, P3, X, and X^+^ emission peaks. All of these results indicate a possible contribution of an additional mechanism for the PL intensity of WSe_2_ for magnetic fields that tune X_*B*_ ^22–24^ into resonance with excitonic states in WSe_2_, such as an RET effect. The schematic in Figure 2(f) provides an overview of the PL of pristine CrSBr and ML-WSe_2_ for B_*z*_ = 0 T and B_*z*_ > B_*z sat*_. The PL energy of X_*B*_ in CrSBr is near the PL energy of pristine ML-WSe_2_. Applying an external magnetic field allows us to tune the X_*B*_ of CrSBr into resonance with different exciton states in WSe_2_ which could modify the PL intensity in the ML-WSe_2_/CrSBr heterostructure.

In order to explore the nature of the sharp PL peaks, we also analyzed the magnetic field dependence of the energy peaks. Figure 2(e) presents a close-up view of the color-coded map of the magnetic field dependence of the PL intensity for the WSe_2_/CrSBr heterostructure in the range of sharp PL peaks (labeled P1, P2, P3, P4, and P5) and their extracted *g*-factor values (details in Figure S11 and equation (S1) in the SI). We observe clear evidence of doublet structures for several sharp PL peaks in agreement with our interpretation of DDE.^36,42,48^ Additionally, the extracted *g*-factor values of these sharp peaks are |*g*| ≈ 9, also in agreement with this interpretation.^34,36^ Moreover, we remark that the circular polarization degree (CPD) of the bright X and X^+^ and sharp emission peaks have opposite signs (Figure 2(d)), which suggests that they could be related to localized positively charged dark trions.^60–63^ In addition, the CPD for all emission peaks (Figure S10) shows important changes under the RET condition.

To obtain a deeper understanding of the properties of our heterostructure, we have additionally measured magneto-PL with the laser excitation polarized along the â axis (Figure S14). Under this condition, the PL intensity of CrSBr is much weaker due to the anisotropic properties of CrSBr. Similar results were also observed, which are consistent with a possible RET effect. In general, these experimental results indicate a possible contribution of a magnetic field-controlled interlayer RET effect involving the X_*B*_ in CrSBr and excitonic states in WSe_2_ in the heterostructure. Figure 2(h) shows a schematic drawing of the RET tuned by the magnetic field in the ML-WSe_2_/CrSBr heterostructure. Depending on the magnetic field, the RET effect modifies the PL peak in ML-WSe_2_.

There are different types of RET, such as Förster^64,65^ and Dexter coupling.^66^ Both of them were observed in several systems including type-II TMD vdWHs.^64–66^ However, there is no previous report on energy transfer in vdWH’s composed of TMD and magnetic 2D materials. In particular, the Förster-type coupling occurs only with bright emissions which have in-plane dipole momentum,^64,65^ and it is limited to a length scale of <10 nm. As dark excitons have out-of-plane dipole momentum,^67^ they do not allow an efficient Förster-type RET effect. However, weakly localized defective dark excitons in ML-WSe_2_, usually observed at around 1.68 eV, have an in-plane dipole moment which could favor the RET effect^67^ in the WSe_2_/CrSBr heterostructure. On the other hand, for a Dexter type of RET effect, there is no requirement to have bright emissions as it is not related to the dipole interactions. Actually, Dexter-type RET depends on the overlap of their electron wave functions and therefore requires that the two layers must be closely contacted (<1 nm).^65–68^ In particular, Förster-type RET would be more likely, since it is a dipole–dipole coupling and is independent of the spin direction. Dexter-type RET relies on the wave function overlap, which is expected to be more suppressed because of the perpendicular spin directions in both materials. However, as it was previously reported that the magnetization direction of CrSBr has a perpendicular component^32^ due to magnetic proximity effects in a TMD/CrSBr heterostructure, it could allow a possible contribution of Dexter-type RET for the WSe_2_/CrSBr heterostructure.

In order to understand our results in more detail, we also performed time-resolved PL (TRPL) measurements (Figure S23 in the SI file). We observed a reduction of the PL time decay in the resonant energy condition. For a Förster-type RET, the acceptor should have a longer decay time. However, the assignment of which material is the acceptor and which is the donor in our heterostructure is not straightforward. In addition, we also prepared a sample CrSBr/hBN/WSe_2_/hBN (Figure S21) with a thin layer of hBN (of about 2 nm) between WSe_2_ and CrSBr. We observed that both effects (charge transfer and RET) were suppressed (Figure S22), which indicates that they occur at a short distance (<2 nm). This result could suggest that the RET effect is strongly dependent on the overlap of the wave functions of carriers in the WSe_2_ and CrSBr layers, as expected for Dexter-type RET. However, the detailed mechanism for the RET in WSe_2_/CrSBr is still unknown. Therefore, additional studies are necessary to fully understand the nature of RET in the WSe_2_/CrSBr heterostructure.

We have also performed circular polarization-resolved PL measurements under parallel magnetic fields B⃗∥b̂ (B_*y*_). Figure 3(a,b) shows the color-coded map of the PL intensity as a function of B_*y*_ for X_*A*_ and X_*B*_ in a pristine CrSBr flake showing the magnetic field-induced phase transition of CrSBr at around |*B*_*y sat*_| ≈ 0.375 T. Figure 3(c) presents the typical PL spectra for X_*B*_ in CrSBr showing the higher red shift of the PL band after the CrSBr magnetic field phase transition.

**Figure 3:**
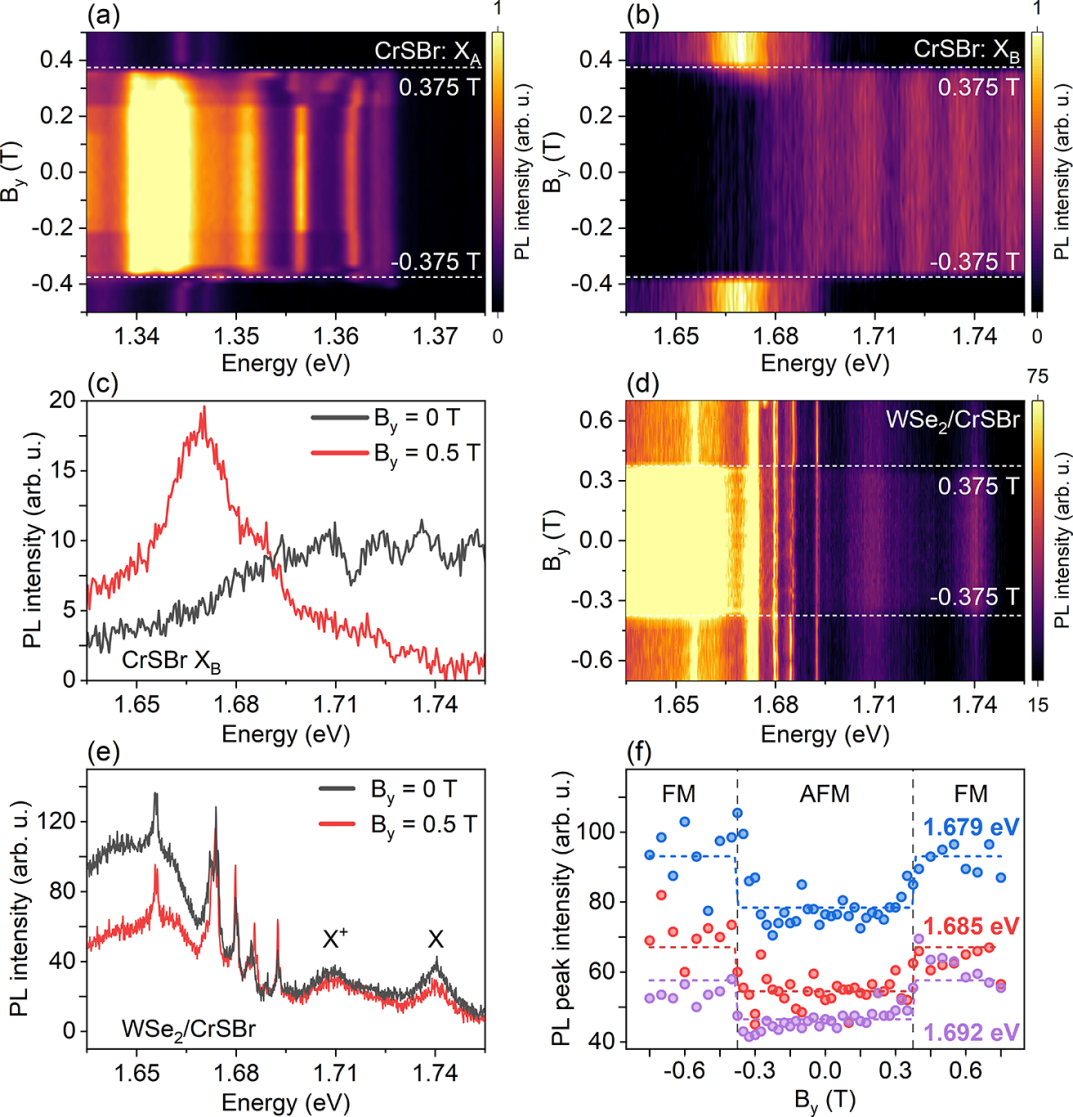
(a,b) Color-coded map of the circularly polarized PL intensity of the A and B excitons in the pristine CrSBr layer as a function of magnetic field applied along the b̂ axis (labeled B_*y*_). (c) Typical PL spectra of the B exciton of CrSBr before and after its magnetic phase transition. (d) Color-coded map of the circularly polarized PL intensity of WSe_2_/CrSBr as a function of the parallel magnetic field. (e) PL spectra of WSe_2_/CrSBr before and after the magnetic phase transition of CrSBr. (f) Integrated PL intensity of selected DDE PL peaks in WSe_2_/CrSBr as a function of the magnetic field. The PL data were obtained using a laser with energy 1.88 eV linearly polarized along the b̂ axis and at 3.6 K.

Figure 3(d) presents the color-coded map of the PL intensity for ML-WSe_2_/CrSBr as a function of magnetic field. Remarkably, the PL intensity of some sharp emission peaks shows an abrupt enhancement while the other PL peaks, which are not resonant with the X_*B*_, show an abrupt reduction in PL intensity for B_*y*_ > B_*y sat*_. This PL intensity enhancement is illustrated in the selected PL sharp peaks before and after the magnetic phase transition (Figure 3(e)) and also in the magnetic field dependence of the PL intensity of these sharp PL peaks (Figure 3(f)) in WSe_2_/CrSB. In contrast to the condition of the out-of-plane magnetic field, the exciton/trion peaks also show a decrease in PL intensity. This occurs because under a parallel magnetic field there is no resonance condition for trion/exciton and X_*B*_ and the PL intensity is dominated by charge transfer effects. This PL intensity enhancement is associated with a possible contribution of the RET effect while the PL decrease is associated with changes in the degree of charge transfer.

To shed more light on the impact of the anisotropic properties of the CrSBr material on the RET, we also performed linear polarization-resolved PL measurements under a parallel magnetic field before and after the field-induced ferromagnetic state of the CrSBr. The use of anisotropic materials is expected to provide additional degrees of freedom for the directional control of the RET effect in vdWH’s.^69^ Figure 4(a,b) presents color-coded maps of the linearly polarized PL intensity as a function of the angle of in-plane linear polarization under 0 T and −1 T at 3.6 K (B⃗∥b̂) . Figure 4(c,d) shows the PL spectra under 0 T and −1 T for linear polarization detection along the â axis (90°) and b̂ axis (0°) of CrSBr. We clearly observe that the CrSBr PL peaks are strongly linearly polarized along the b̂ axis as expected due to the anisotropic properties of CrSBr.^6,9,21^ Interestingly, we also observed that the PL intensity of the emission peaks in WSe_2_/CrSBr is dependent on the in-plane linear polarization angle. Moreover, we also observed a clear enhancement of the PL intensity of the sharp emission peaks after the magnetic phase transition of CrSBr (|*B*_*y*_| > 0.375 T) and only for linear polarization detection along the b̂ axis probably due to the anisotropic properties of CrSBr. This result is also consistent with our interpretation of the RET effect to explain the brightening of sharp emissions. In general, our findings suggest an anisotropic RET effect which could dominate the charge transfer effect for this WSe_2_/CrSBr heterostructure.

**Figure 4:**
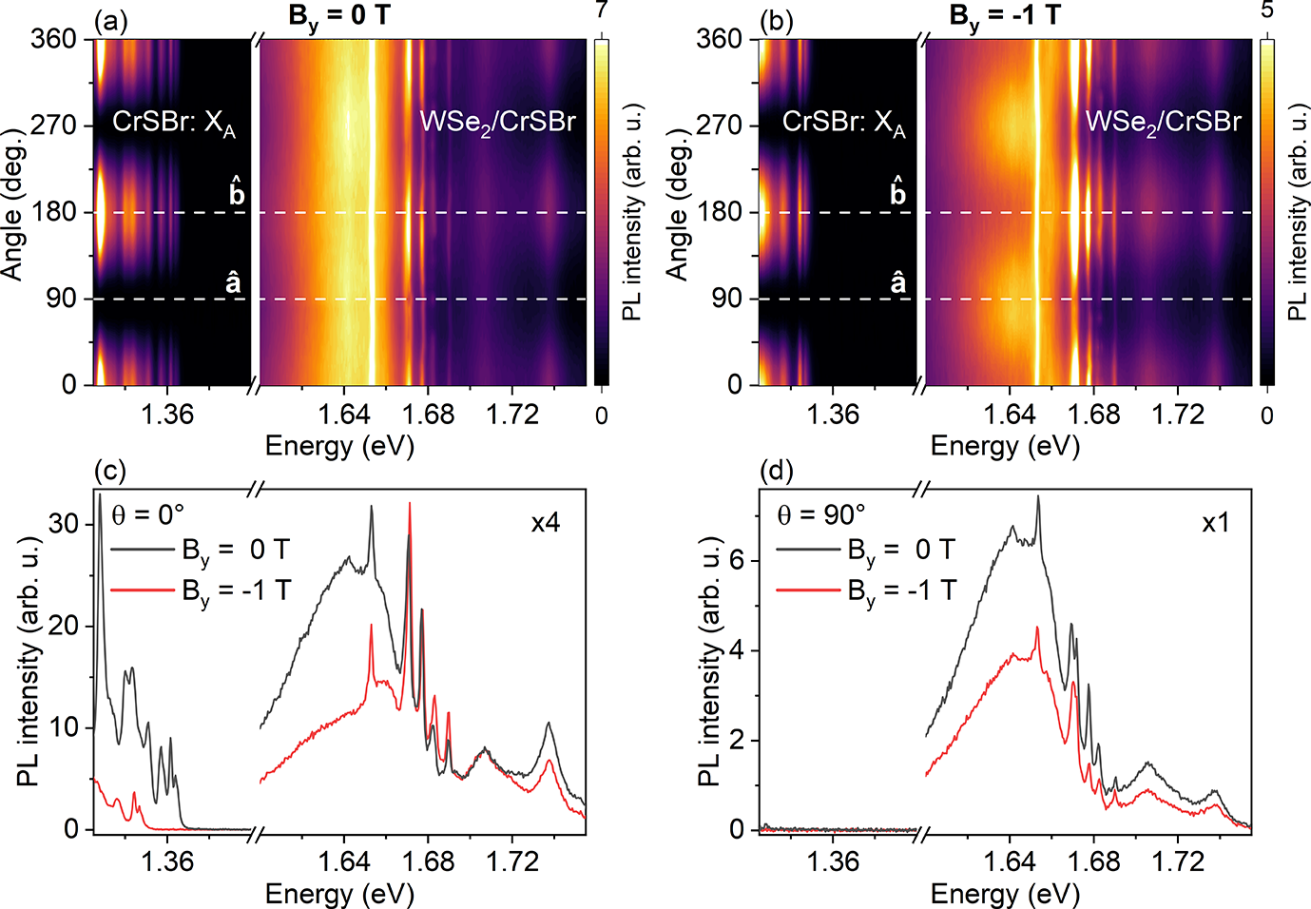
(a,b) Color-coded map of the linearly polarized emission intensity as a function of the angle of in-plane linear polarization for the WSe_2_/CrSBr heterostructure at 0 T and after the magnetic phase transition (−1 T), respectively, at 3.6 K. The magnetic field was applied along the b̂ axis of CrSBr (B_*y*_). (c,d) PL spectra at 0 T and −1 T for polarization angles of 0 and 90°, respectively, which suggests a possible anisotropic RET effect. The laser is linearly polarized along the b̂ axis.

In summary, we investigated excitonic properties in a WSe_2_/CrSBr heterostructure using linearly and circularly polarized photoluminescence under parallel and out-of-plane magnetic fields. Our results demonstrate important modifications in the excitonic properties of ML-WSe_2_ under an increasing magnetic field induced by the adjacent CrSBr layer. A clear PL intensity enhancement is observed for emission peaks in WSe_2_, under external magnetic fields that tune the X_*B*_ in CrSBr into resonance with emission peaks in WSe_2_. This effect is associated with a possible contribution of an RET effect, controlled by the magnetic field and involving excitonic states in CrSBr and ML-WSe_2_. On the other hand, for ML-WSe_2_ PL peaks that are not in resonance, a reduction in the WSe_2_ PL intensity is observed after the field-induced FM order of CrSBr. This effect is associated with a change in the degree of charge transfer after the magnetic field-induced phase transition of CrSBr. Furthermore, we show that this PL enhancement is anisotropic and has a short-range interaction (<2 nm). However, further studies are necessary to understand in detail the nature of the different mechanisms in the WSe_2_/CrSBr heterostructure. Our findings underscore that the magnetic control of the RET could be a useful tool to modify the optical properties of 2D materials in anisotropic magnetic vdWH’s. The appropriate design of magnetic vdWH’s could be an interesting platform for the engineering of new devices for possible applications in optoelectronics, optospintronics, and quantum technology.

## Supplementary Material



## Notes

Details of sample preparation and complementary low-temperature PL and magneto-PL results for different laser positions (PDF)
