# Supplementary material for: Interplay of Energy and Charge Transfer in WSe2/CrSBr Heterostructures
Source: Nano Lett. 2025 Aug 22;25(35):13212–20. doi: 10.1021/acs.nanolett.5c03150 (PMC12412143; doi:10.1021/acs.nanolett.5c03150)
Supplement: Supplementary file 1 [file nl5c03150_si_001.pdf]

Supplementary Information: Interplay of energy and charge transfer in WSe_2/CrSBr heterostructures

José Roberto de Toledo,[*,†] Caique Serati de Brito,[†] Barbara L. T. Rosa,[‡] Alisson R. Cadore,[¶,§] César Ricardo Rabahi,[†] Paulo E. Faria Junior,[∥,⊥] Ana Carolina Ferreira de Brito,[#] Talieh S. Ghiasi,[@] Josep Ingla-Aynés,[@] Christian Schüller,[△] Herre S. J. van der Zant,[@] Stephan Reitzenstein,[‡] Ingrid D. Barcelos,[#] Florian Dirnberger,[∇,††,‡‡] and Yara Galvão Gobato[*,†]

†*Physics Department, Federal University of São Carlos, 13565-905 São Carlos, SP Brazil*

‡*Institut für Festkörperphysik, Technische Universität, 10623 Berlin, Germany*

¶*Brazilian Nanotechnology National Laboratory (LNNano), Brazilian Center for Research in Energy and Materials (CNPEM), 13083-100 Campinas, SP, Brazil*

§*Programa de Pós-Graduação em Física, Instituto de Física, Universidade Federal do Mato Grosso, 79070-900 Cuiabá , Brazil*

∥*Department of Physics, University of Central Florida, Orlando, 32816 Florida, USA*

⊥*Department of Electrical and Computer Engineering, University of Central Florida, Orlando, 32816 Florida, USA*

#*Brazilian Synchrotron Light Laboratory (LNLS), Brazilian Center for Research in Energy and Materials (CNPEM), 13083-100 Campinas, SP, Brazil*

@*Kavli Institute of Nanoscience, Delft University of Technology, 2628 CJ Delft, The Netherlands*

△*Institut für Experimentelle und Angewandte Physik, Universität Regensburg, D-93040 Regensburg, Germany*

∇*Department of Physics, TUM School of Natural Sciences, Technical University of Munich, 85748 Garching, Germany*

††*Zentrum für Quantum Engineering (ZQE), Technical University of Munich, 85748 Garching, Germany*

‡‡*Munich Center for Quantum Science and Technology (MCQST), Technical University of Munich, 85748 Garching, Germany*

E-mail: jose.toledo@df.ufscar.br; yara@ufscar.br

Materials and methods

The WSe_2/CrSBr heterostructures were prepared using conventional all-dry transfer techniques and consist of a WSe_2 monolayer onto a CrSBr bulk covered by a thin layer of hexagonal boron nitride (hBN). The WSe_2 crystal was purchased from 2D Semiconductors, while the hBN and CrSBr crystals were obtained from HQ Graphene. The WSe_2 monolayers, hBN thin layers, and CrSBr flakes were obtained through mechanical exfoliation using Scotch tape, followed by direct transfer onto clean SiO_2/Si substrates. The hBN layer was picked up using a polycarbonate (PC) film at 70 °C, and the hBN was then used to pick up the WSe_2 monolayer. The hBN/WSe_2 stack was subsequently transferred onto a CrSBr flake. Following this, the final stack was heated to 180 °C for less than a minute to melt the PC covering, which was then removed from the sample's surface by dissolving it in chloroform. After preparation, the WSe_2/CrSBr heterostructure was placed in a vacuum chamber (pressure of approximately 6×10^{-5} mbar) and slowly heated to 150 °C at a rate of 1 °C/min, remaining at this temperature for 4 hours.

The Atomic Force Microscopy (AFM) experiments were carried out on a NX10 scanning probe microscope (Park Systems) using the intermittent contact imaging mode with PPP-FMR (Nanosensors) probes (ω_0 = 75 kHz; k = 2.8 N·m^{-1}). The measurements were performed under ambient temperature and controlled humidity conditions (relative humidity below 5%, with a N_2 flow). All AFM images were processed by using the Gwyddion software.

Figure S1(a) shows the AFM image of the CrSBr/WSe_2/hBN heterostructure. The CrSBr flake in the region of the WSe_2/CrSBr has a thickness of approximately 70 nm (red curve in Figure S1.b). Several nano-bubbles of different sizes are observed on the WSe_2/CrSBr heterostructure (blue curve in Figure S1 (b)).

Photoluminescence excitation experiments (PLE) were performed using a closed-cycle cryostat (Attocube - Attodry 800) with temperatures up to 3.6 K. The photoluminescence was measured using an optical parametric oscillator (OPO) laser (80 MHz repetition rate, APE - picoEmerald) and objective lens 60x (NA = 0.81). The PL signal was dispersed by

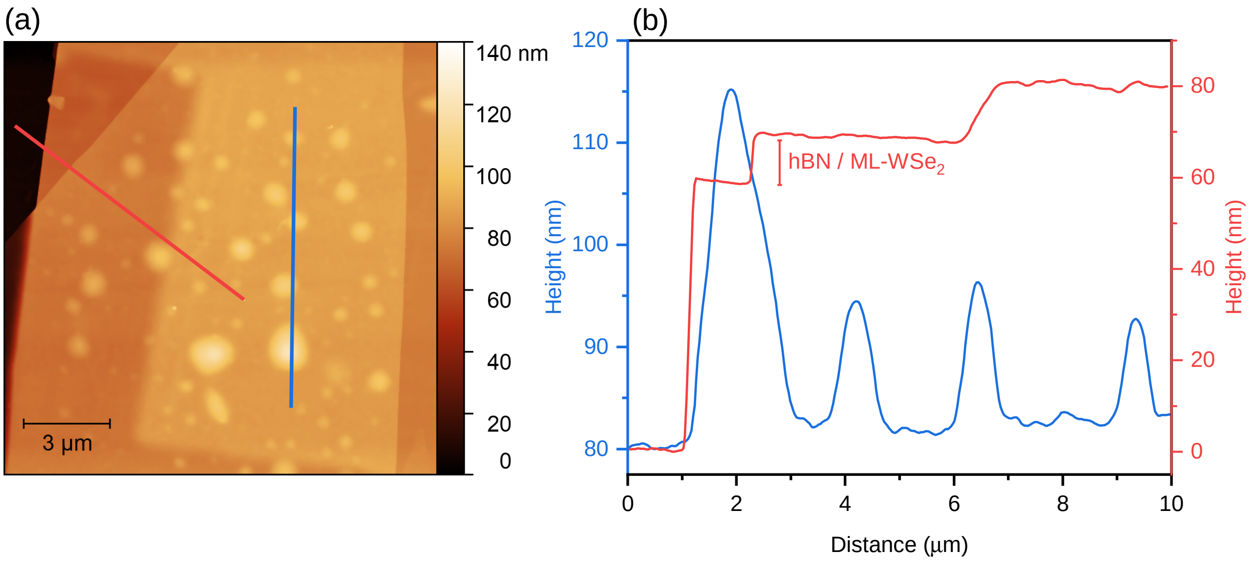

Figure S1: (a) AFM topography image of the CrSBr/WSe_2/hBN heterostructure (15×15 μm^2) on a SiO_2/Si substrate using the tapping mode. (b) Height profiles of the heterostructure measured along the red and blue lines in panel (a).

a 75 cm spectrometer (SpectraPro HRS-750) with a 300 l/mm grating and detected with a CCD camera (Teledyne Princeton - PIXIS).

Magneto-photoluminescence measurements were performed at a temperature of 3.6 K in a closed-cycle cryostat equipped with superconducting magnet coils under magnetic fields up to 9 T (Attocube - Attodry 1000). The sample was mounted on an x-y-z piezoelectric Attocube stage. A continuous-wave (cw) linearly-polarized laser with a photon energy of 1.88 eV was used to excite the sample. The photoluminescence signal was collimated using an aspheric lens (Attocube LT-IWDO, NA = 0.68), and the selection of circular polarization components was performed before focusing onto a 50 μm multimode optical fiber. The signal was then dispersed by a 75 cm spectrometer with gratings of 150 l/mm or 600 l/mm and detected by a silicon CCD detector (Andor, Shamrock/iDus).

Time resolved PL measurements (TRPL) were performed using a standard technique TCSPC (PicoQuant / PicoHarp 300) with a 660 nm pulsed laser (APE-Berlin / PicoEmerald) delivering pulses of 2 ps at a repetition rate of 80 MHz. The PL signal were dispersed by a 75 cm spectrometer and detected by a PMA hybrid device (PMA Hybrid 50, PicoQuant).

Additional Results

WSe_2/CrSBr heterostructure

Recent DFT calculations[1,2] have shown that the $MoSe_2$/CrSBr heterostructure displays a type-III band alignment with the valence band (VB) of $MoSe_2$ above the conduction band (CB) of CrSBr. Since the band alignment of $MoSe_2$ and WSe_2 is already known to be of type-II (the VB of WSe_2 is above the VB of $MoSe_2$)[3,4], the WSe_2/CrSBr heterostructure should also exhibit a type-III band alignment with the VB of WSe_2 even more inside the CB of CrSBr, suggesting a stronger p-type doping regime for WSe_2, as depicted in Figure S2.

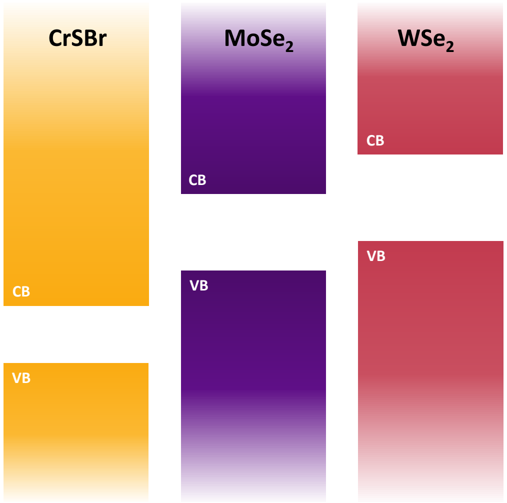

Figure S2: Schematic representation of the band alignments for the CrSBr, $MoSe_2$ and WSe_2 layers.

Optical properties of WSe_2/CrSBr heterostructure

Figures S3 (a) and (b) show the typical laser power dependence of the PL spectra of WSe_2/CrSBr at 3.6 K (for the laser position S2 indicated in Figure 1 (d)). As the heterostructure shows the presence of several nano-bubbles with diameter below 1 μm and heights up to 35 nm (Figure S1) and defect states are usually found in the layer, the observed sharp emission peaks are associated with defective dark exciton (DDE) states[5–8], due to the hybridization of strain-localized dark excitons with defect states in ML-WSe_2. The

nature of these defect states is usually associated to the presence of Se vacancies or impurities[5–8]. The Figures S3 (c) and S3 (d) show the integrated PL intensities of the defective dark excitons (DDE) (peaks P1, P2, P3, P4), bright exciton (X) and trion (X^+). The fitted curves were obtained using $I = P^\beta$, where I is the integrated PL intensity and P is the laser power. For non-localized excitons, a linear behavior with $\beta \approx 1$ is expected. The extracted β-values for both X and X^+ are around 0.94 and 0.96, respectively as expected for non-localized emissions. However, for DDE peaks a sub-linear behavior ($\beta \leq 0.4$) is observed, suggesting that these peaks are localized excitons.

Figure S4 shows the temperature dependence of the PL spectra of the DDE peaks in the WSe_2/CrSBr heterostructure (for the laser position S5 indicated in Figure 1 (d)). We remark that these sharp PL peaks were observed only at low temperatures (T $<$ 25 K) in agreement with previous results in the literature[6,7].

Figure S5 illustrates the typical time fluctuation effects for the laser position S2 over a short timescale (about 83 min), showing the jittering effects of the DDE PL peaks, i.e., typical fluctuations in the energies and intensities of these PL peaks.

Figure S6 shows the color-coded map of the PL intensity as a function of excitation energy for a typical DDE PL peak. We observed that the PL intensity is stronger for laser energy excitation around the WSe_2 exciton PL peak (X) in WSe_2/CrSBr heterostructure.

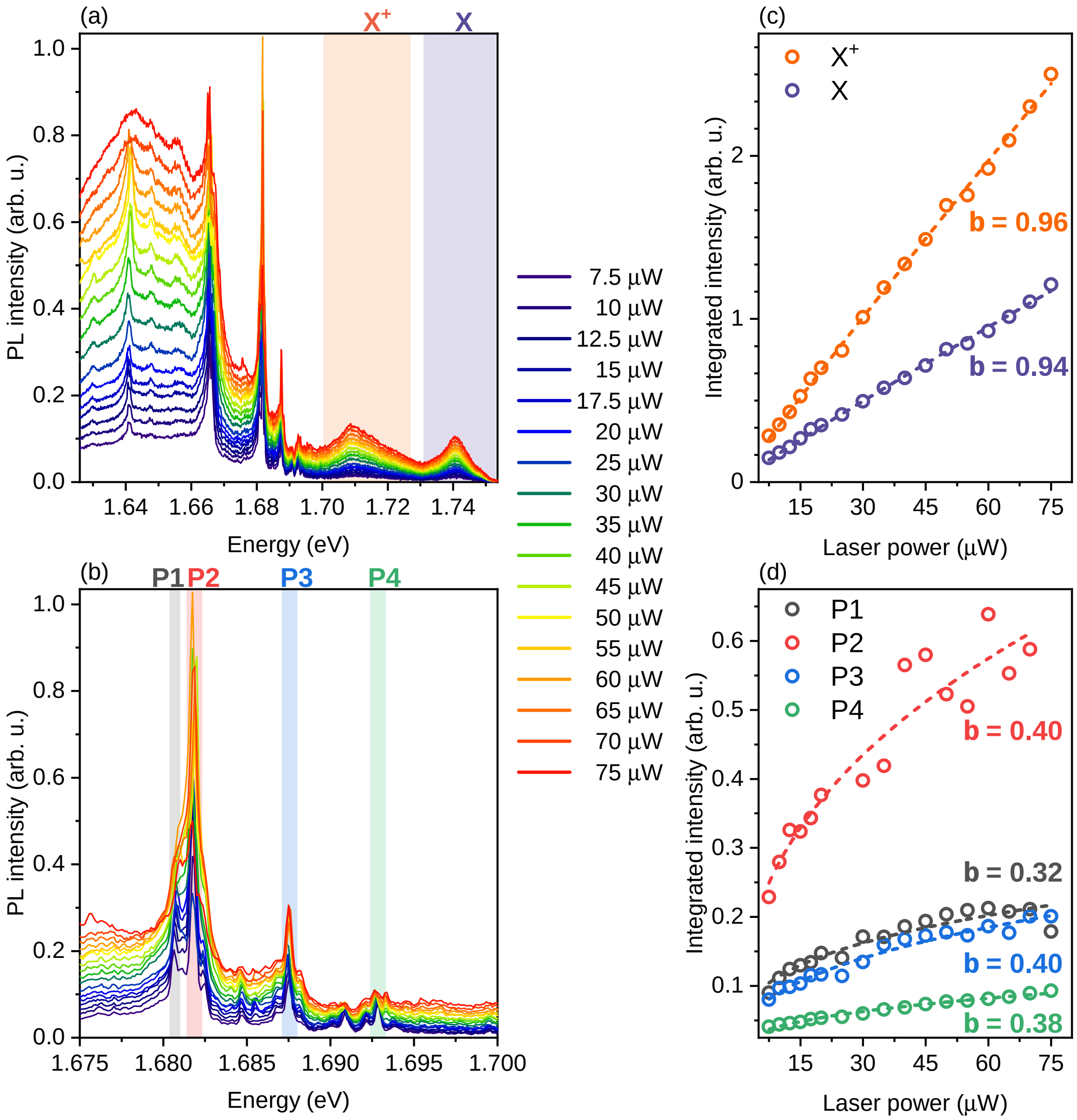

Figure S3: (a) Laser power dependence of the PL spectrum for different emission peaks in WSe_2/CrSBr and (b) Enlarged view of the laser power dependence for the energy range corresponding to the emission of the DDE peaks. (c) and (d) show the integrated PL intensities as a function of laser power for X and X^+, and for DDE peaks, respectively. The dashed lines represent the fitting curves obtained using $I = P^{\beta}$. The PL spectra were measured for the laser position S2 using a 600 l/mm grating.

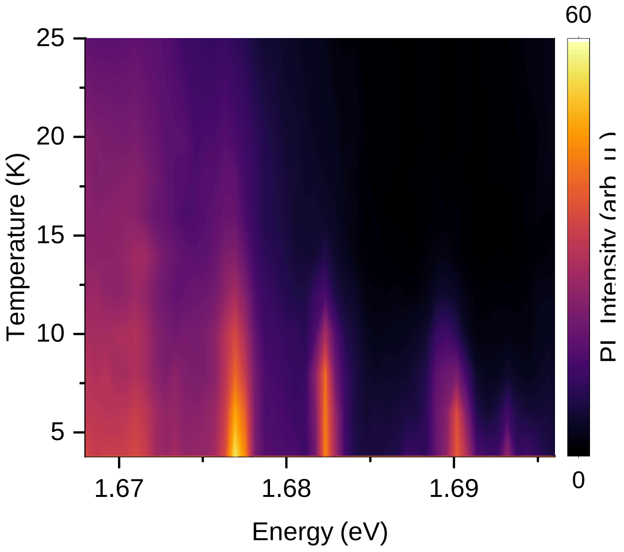

Figure S4: Color coded map of the temperature dependence of the PL intensity of the DDE peaks. The PL spectra were collected for laser position S5 and using a grating of 300 l/mm.

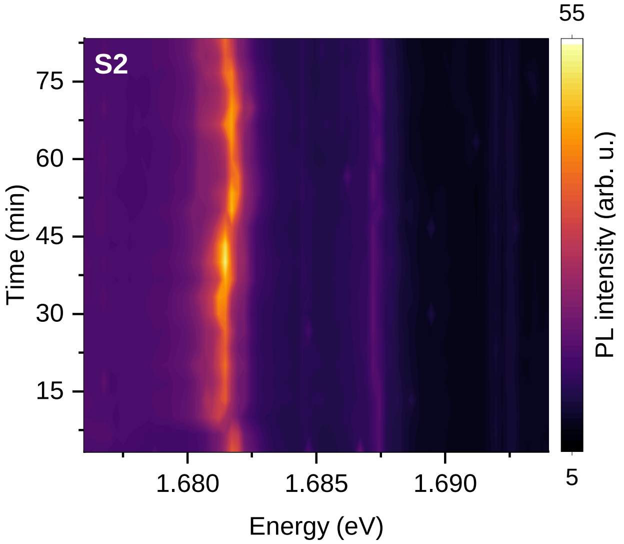

Figure S5: Color-coded map of the circularly-polarized PL intensity of the DDE peaks as a function of time at 3.6 K.

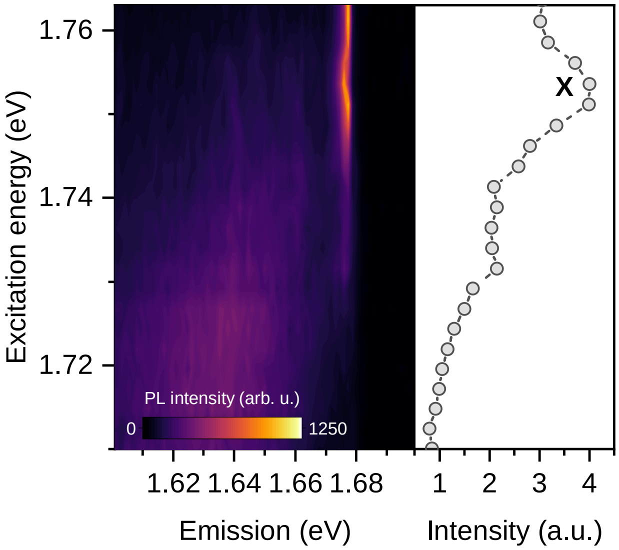

Figure S6: Color-coded map of the PL intensity of the WSe_2/CrSBr as a function of the laser excitation energy at 3.6 K. The lateral inset shows the integrated PL intensity of the DDE peak at 1.677 eV. The PL signal was dispersed by a grating of 300 l/mm.

Optical and magneto-optical properties of pristine CrSBr

Figure S7 shows the PL spectra of the X_B in CrSBr at different sample positions on the CrSBr pristine layer, for both the AFM (Figure S7(a)) and FM (Figure S7(b)) orders of CrSBr. They consist predominantly of two broad bands. The PL spectra in Figure S7(a) were best fitted using two Voigt profiles centered at approximately 1.674 eV and 1.737 eV, with linewidth of about 30 meV, while in the Figure S7(b), they were fitted using Voigt profiles centered at approximately 1.668 eV (FWHM $\approx$ 5 meV) and 1.687 eV (FWHM $\approx$ 12 meV). Broad background peaks were also included to minimize the residuals. For the PL measurements at $B = 0$ T, a 50$\times$ objective lens (NA = 0.55) and a CW 633 nm laser excitation were used in a Witec Raman/PL system coupled with a Montana cryostat.

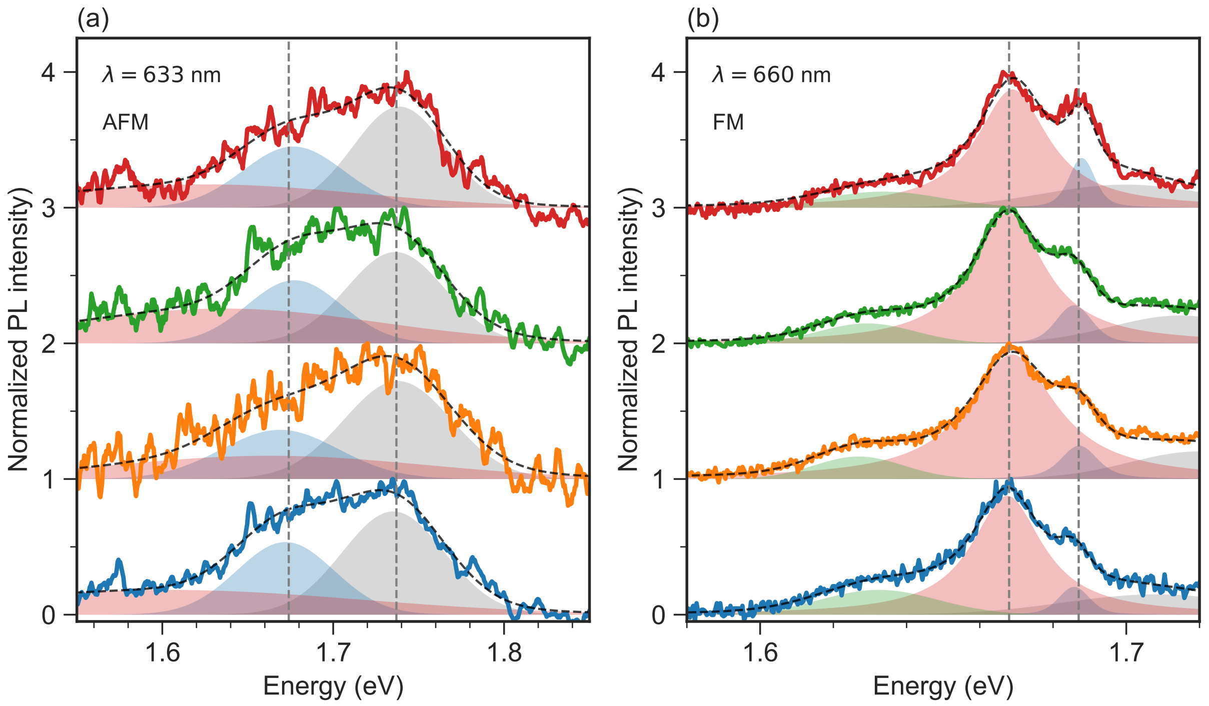

Figure S7: PL spectra of X_B in CrSBr at different sample positions at 3.6 K under (a) 633 nm excitation at B = 0 T (in AFM order) and (b) 660 nm excitation in the FM order. The shaded areas in each panel represent Voigt profiles obtained by fitting the PL spectra, with the average energy of the dominant peaks indicated by vertical dashed lines.

Figure S8 shows the laser power dependence of the PL intensity of X_B in CrSBr under applied perpendicular magnetic field (3T). We observed that the relative intensity of the P1 and P2 bands depends on the laser power value (Figure S8(b)).

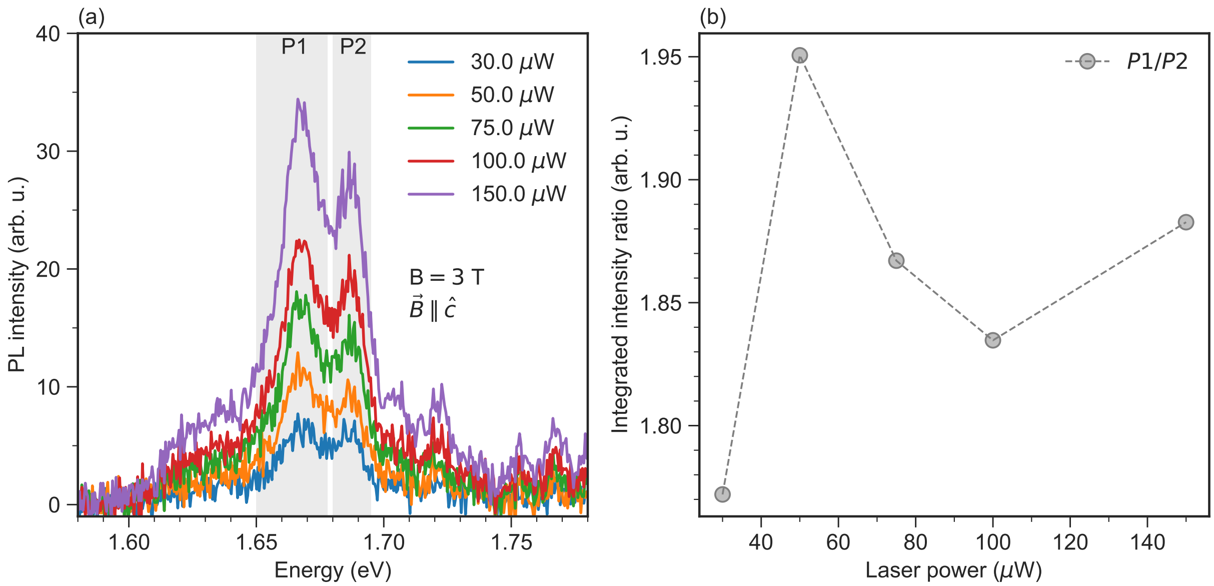

Figure S8: (a) Laser power dependence of PL spectra of X_B in CrSBr under 3T (magnetic field applied along the crystallographic $\hat{c}$-axis). (b) Relative PL intensity of P1 and P2 bands as a function of laser power. The PL spectra were measured using linearly polarized 660 nm laser excitation at 3.6 K.

Figure S9 shows the magnetic field dependence of the PL peak energy for the fundamental (X_A) and a higher energy exciton in CrSBr bulk under parallel and perpendicular magnetic fields. Particularly, as we have used a laser photon energy around a resonant absorption of CrSBr, the PL signal of the X_B is enhanced as compared to the experimental condition of higher energy photon laser excitation of previous works in the literature[9–12]. For both A and B excitons, the PL peak energy shows a redshift with saturation fields of $|B_{z\ sat}| \approx 2.2$ T and $|B_{y\ sat}| \approx 0.375$ T for the applied magnetic field along the hard $\hat{c}$-axis (Figures S9(a) and (c)) and easy $\hat{b}$-axis (Figures S9(b) and (d)), respectively.

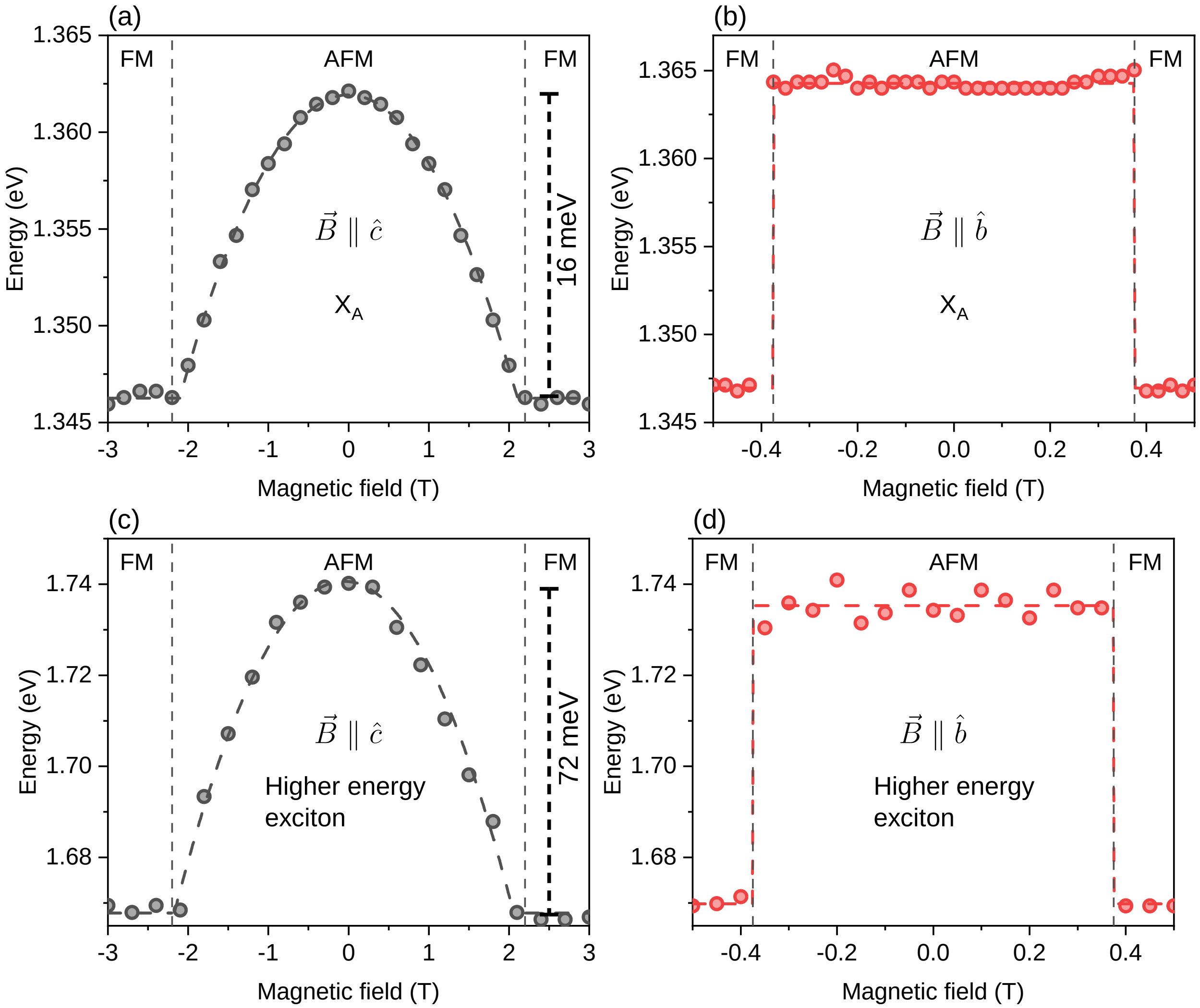

Figure S9: (a, b) PL peak energy of X_A in CrSBr as a function of the magnetic field applied along the $\hat{c}$ and $\hat{b}$-axis, respectively. (c, d) Magnetic field dependence of the PL peak energy of X_B under magnetic field applied along the $\hat{c}$ and $\hat{b}$-axis, respectively. The vertical dashed lines indicate the saturation magnetic field.

Magneto-optical properties of WSe_2/CrSBr heterostructure

Figure S10 shows the circularly polarized PL spectra for selected magnetic fields applied along the $\hat{c}$-axis at 3.6 K . The σ^- PL spectra were selected for positive magnetic fields. The measurements were performed for the laser position S4 in Figure 2.

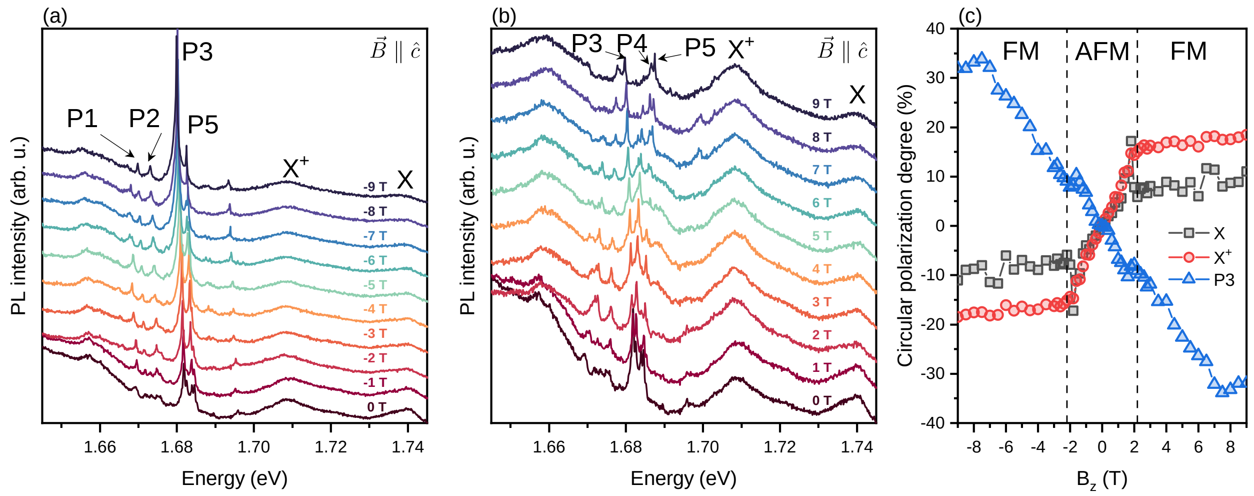

Figure S10: Typical PL spectra of the WSe_2/CrSBr heterostructure for (a) negative and (b) positive perpendicular magnetic fields at 3.6 K. (c) Circular polarization degree of X, X^+ and P3 emissions as a function of magnetic field. The laser is linearly polarized along $\hat{b}$-axis and the PL detection is left circularly polarized for positive magnetic field (σ^-).

The g-factors (g_D) and the fine structure constant δ of the DDE peaks (P1 to P5, Figure S11) were extracted by fitting the magnetic field dependence of the PL peak energy using the equation[13]:

$$E = E_0 \pm \frac{1}{2}\sqrt{\delta^2 \pm (g_D \mu_B B)^2} \tag{S1}$$

where E_0 is the emission peak energy at zero magnetic field, μ_B is the Bohr magneton, and B is the applied magnetic field.

The extracted g-factor values are $|g_D| \approx 9$ and the fine structure constant is $|\delta| \approx 650$ μeV for most of the DDE PL peaks in agreement with previous values reported for localized excitons in ML-WSe_2[13–19].

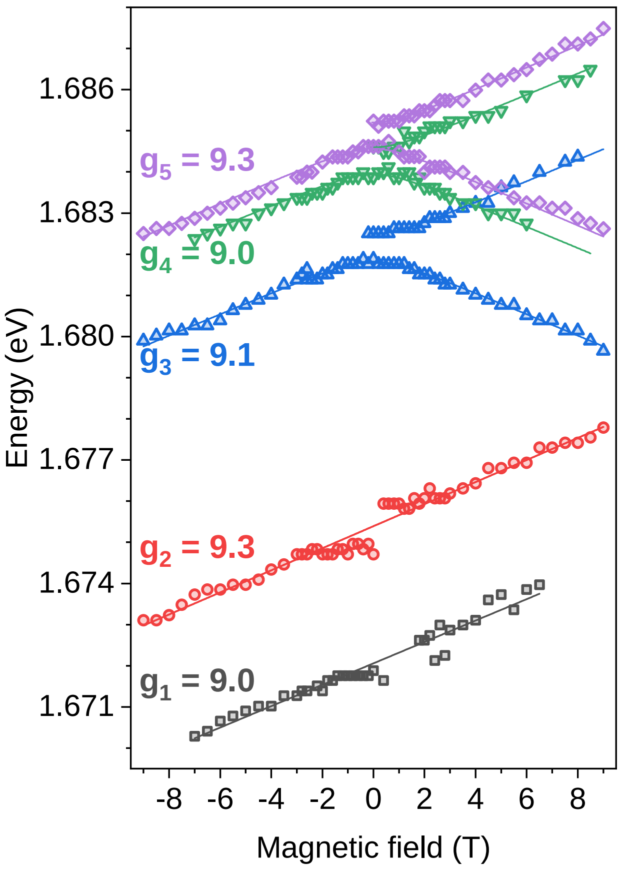

Figure S11: Magnetic field dependence of the DDE PL peak energies. The lines corresponds to the fitting curves using Equation S1.

Figure S12 (a) shows the PL spectra for the exciton peak in WSe_2/CrSBr under perpendicular magnetic fields of -9 T, 0 T and +9 T. We observed a clear asymmetric valley Zeeman shift for positive (9 T) and negative magnetic fields (-9 T). Figure S12 (b) shows the exciton energy peak as a function of the magnetic field. This asymmetric energy shift for positive and negative magnetic fields suggests an asymmetric magnetic proximity interaction. This result is similar to previous results reported for $MoSe_2$/CrSBr heterostructures[1]. Considering time-reversal symmetry, which relates the K^+ and K^- valleys through $g^-(B<0) = -g^+(B>0)$, the exciton g-factor for each valley can be extracted by fitting the exciton energy shift using the following equation[20]:

$$E^{\pm} = E_0 + g^{\pm}\mu_B B \tag{S2}$$

The g-factor values of $g^+ = -1.0 \pm 0.1$ and $g^- = 2.3 \pm 0.1$ were obtained, resulting in a total g-factor of $g = g^+ - g^- = -3.3 \pm 0.1$, which is different from the typical reported values for bright excitons in the pristine WSe_2 monolayer[21–24] ($g \approx -3.8$).

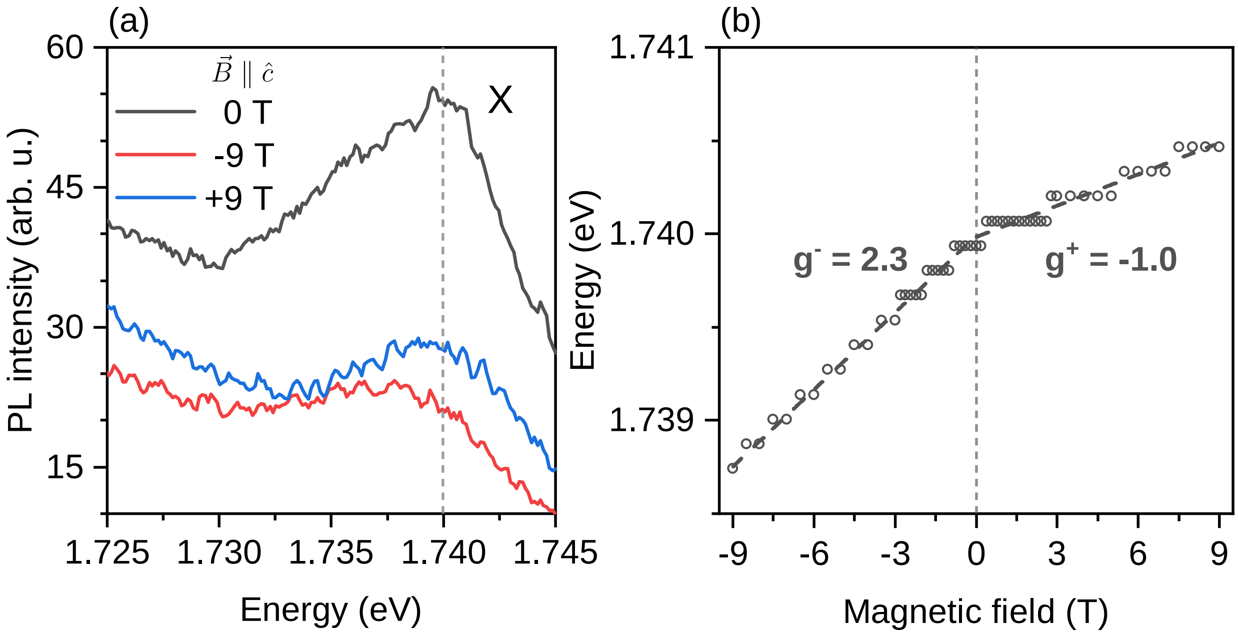

Figure S12: (a) Circularly polarized PL spectra from WSe_2/CrSBr for the selected perpendicular magnetic fields of -9 T, 0 T, and 9 T. (b) Magnetic field dependence of the energy shift of the bright exciton from WSe_2 in the WSe_2/CrSBr heterostructure. The lines correspond to the fitting results using Equation S2. The laser is linearly polarized along $\hat{b}$-axis and the PL detection is left circularly polarized for positive magnetic field.

Figure S13 shows the PL spectra of the WSe_2/CrSBr heterostructure for linearly polarized excitation and left (σ^-) and right (σ^+) circular polarization detection at zero magnetic field, revealing valley-polarized emission of the DDE peaks under zero magnetic field.

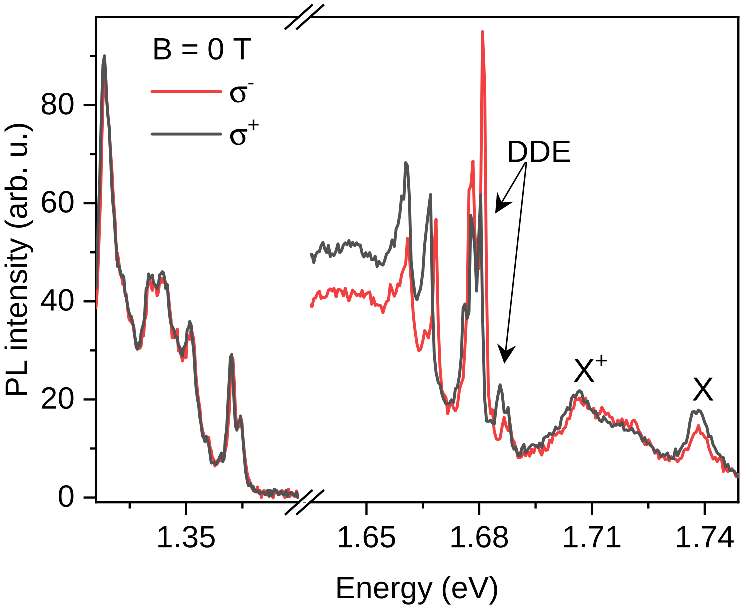

Figure S13: Left (σ^-) and right (σ^+) circularly polarized PL spectra using linearly polarized excitation along $\hat{b}$-axis at 0 T and 3.6 K, demonstrating valley-polarized emission in WSe_2.

Figure S14 shows the color-coded maps of the PL intensity as a function of perpendicular magnetic field for another laser position (position S3 in Figure 1). The sample was excited using linearly polarized laser along the $\hat{a}$ direction, and the σ^+ component was collected for positive magnetic field. For this position, the PL shows a more important jittering effect for the centers of lines. We observed that the PL intensity of X and X^+ of WSe_2 has a different behavior as compared to the results obtained for the linearly polarized laser along the $\hat{b}$ axis, showing an enhancement in its intensity after the magnetic phase transition of CrSBr. However, these results also show a RET effect similar to the results using linearly polarized laser along the $\hat{b}$-axis.

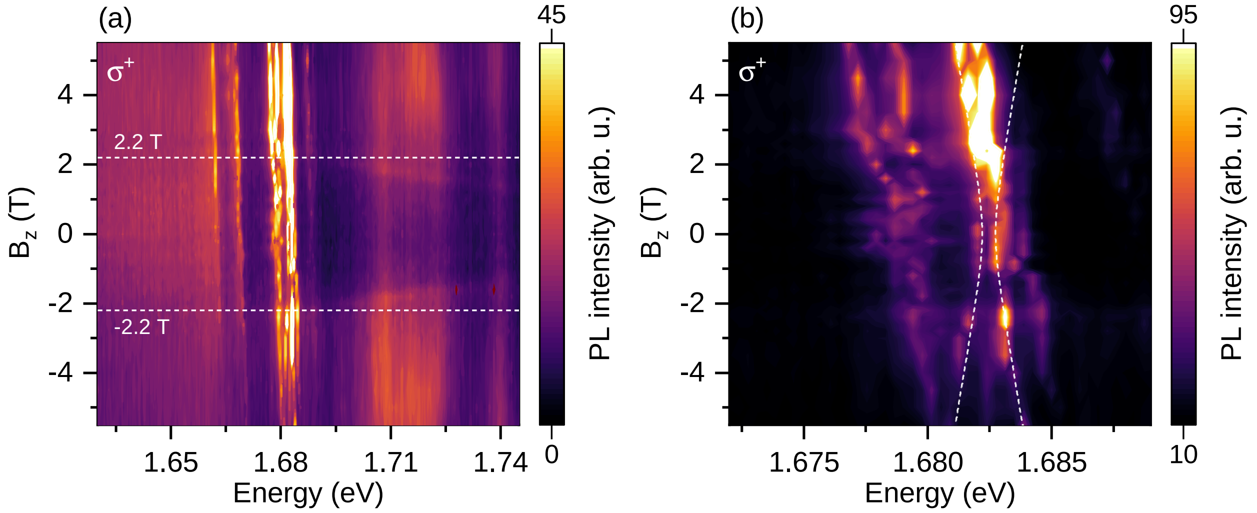

Figure S14: (a) Color-coded map of the PL intensity as a function of magnetic field for the WSe_2/CrSBr heterostructure under $\vec{B} \parallel \hat{c}$, measured at position S3 using a 600 l/mm grating and linearly polarized laser excitation along the $\hat{a}$ direction. (b) Zoom-in of the color-coded map in (a), focusing on the spectral region of the DDE peaks.

Figure S15 shows the magneto-PL results under out-plane magnetic field for another sample position in the WSe_2/CrSBr heterostructure. We observed that the magnetic field dependence of the PL intensity for the exciton/trion peak is also correlated with the magneto PL dependence of X_B in CrSBr (Figure 2), evidencing the reproducibility of the RET effect in the WSe_2/CrSBr system. Figure S15(b) presents the typical PL spectra at this sample position for selected magnetic field values, and Figure S15(c) displays the integrated PL intensities of the X, X^+, DDE, and D bands. An enhancement of the PL intensities for the X, X^+, and DDE bands was observed at $|B_z| = 1.4$ T, $|B_z| = 1.8$ T, and $|B_z| = 2.2$ T, respectively. Despite the broader PL spectra for this sample position, similar features were observed and attributed to the RET effect.

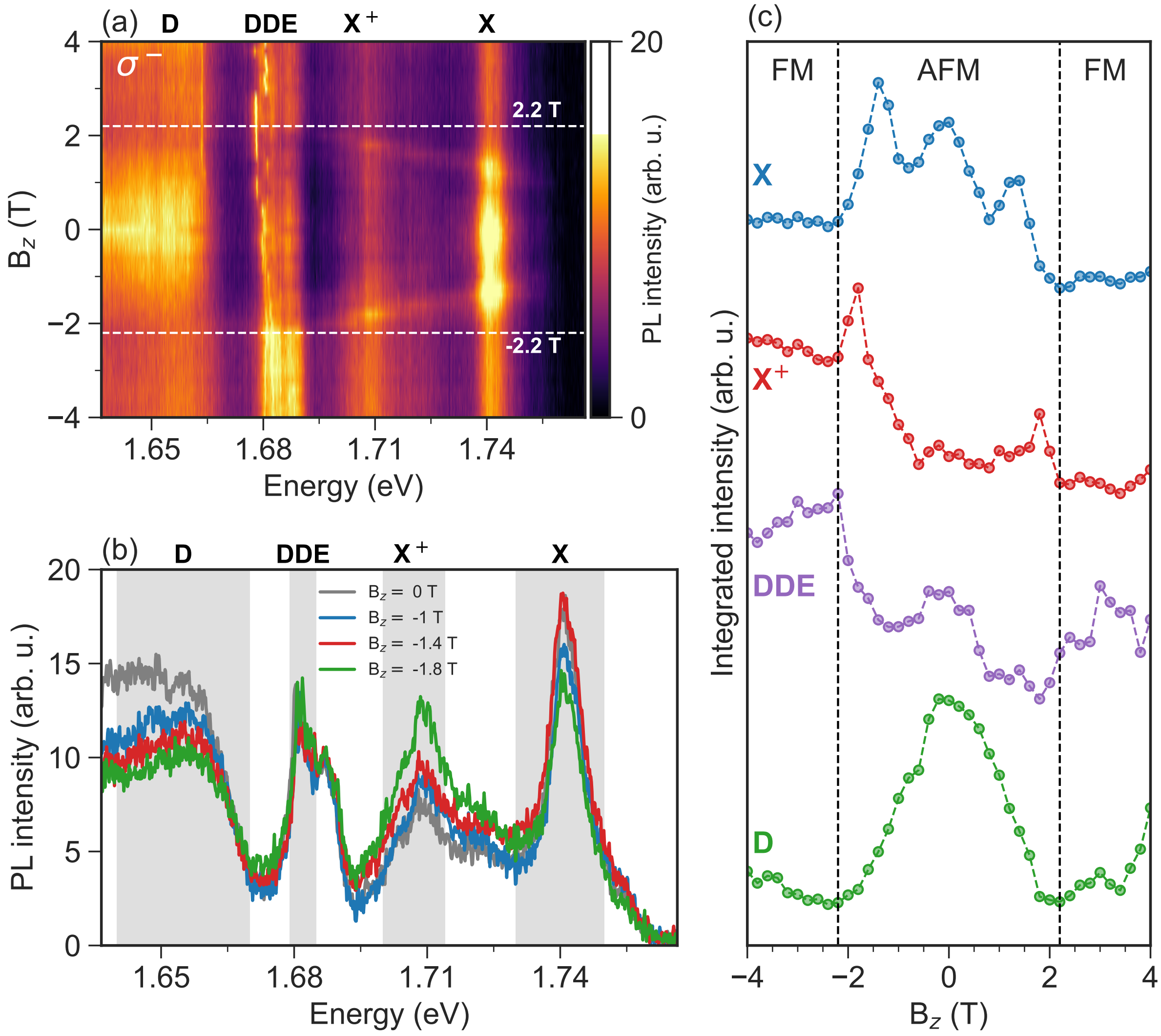

Figure S15: Magneto-PL results using linearly polarized laser excitation along the $\hat{b}$-axis. (a) Color-coded map of the σ^- circularly polarized PL intensity as a function of magnetic field. (b) Selected PL spectra from panel (a). (c) Integrated PL intensity of the X, X^+, DDE, and D emissions. The hatched gray areas in panel (b) indicate the spectral ranges used for integration of each emission band.

Figure S16 shows the linearly polarized emissions in WSe_2/CrSBr as a function of the in-plane polarization angle at 0 T, for linearly polarized excitation along the $\hat{b}$ and $\hat{a}$-axis. It was observed that the X^+ in WSe_2 is linearly polarized and follows the linear polarization of the laser excitation along the two excitation directions. On the other hand, we observed that the X is always polarized around the $\hat{b}$-axis.

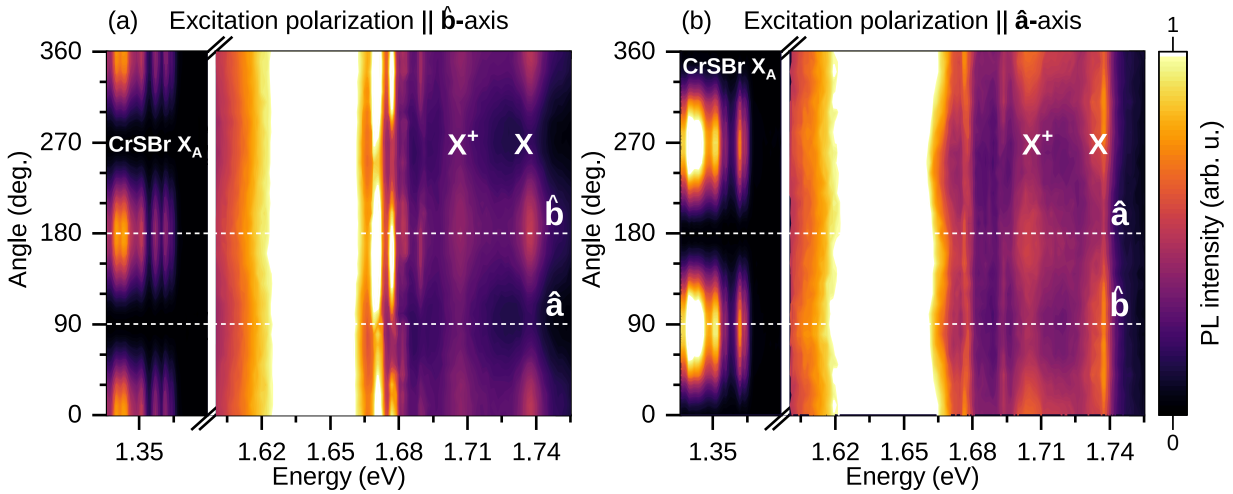

Figure S16: Color-coded maps of the linearly polarized PL intensity of WSe_2/CrSBr for laser excitation linearly polarized along the (a) $\hat{b}$ and (b) $\hat{a}$-axis. Notably, the linearly polarized emission of the X^+ of WSe_2 remains aligned to the excitation direction. The PL spectra were measured at 0 T and 3.6 K, using a grating with 150 l/mm.

Figure S17(a) shows the relative integrated PL intensity of X^+ and X as a function of an applied parallel magnetic field along the crystallographic $\hat{b}$-axis. We observed an abrupt change in relative intensities with increasing magnetic field which evidences a change of doping of WSe_2 after the magnetic field induced FM order of CrSBr. This effect is explained by a change in the degree of charge transfer in the WSe_2/CrSBr heterostructure due to the change of CrSBr band gap in the magnetic field induced FM order in CrSBr.

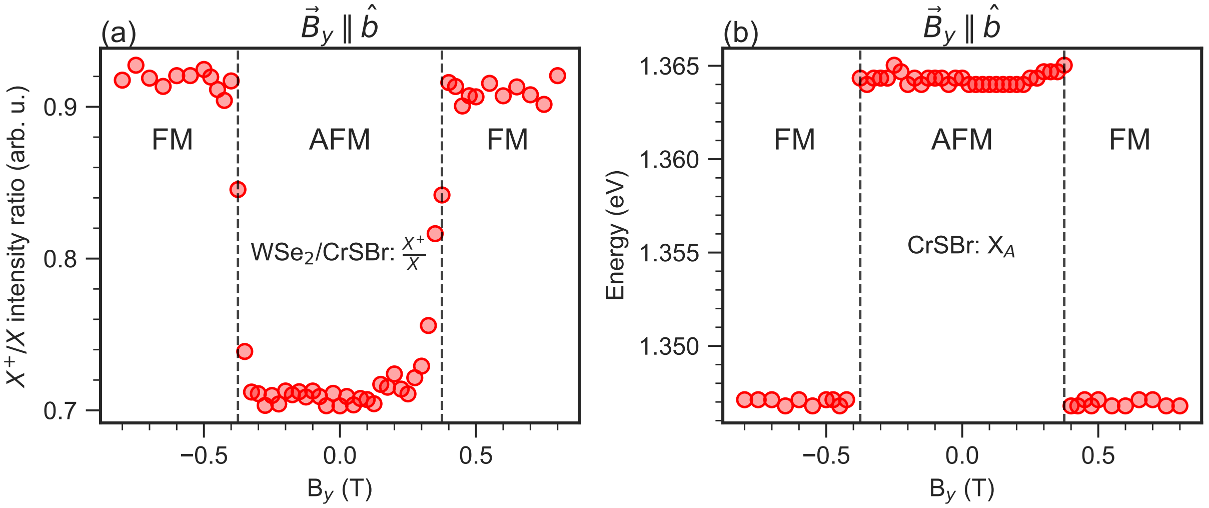

Figure S17: (a) Relative integrated PL intensity X^+/X as a function of an in-plane magnetic field. (b) Magnetic field dependence of X_A in CrSBr along the crystallographic $\hat{b}$-axis. The vertical dashed lines indicate the critical fields ($|B_y| = 0.375$ T) where the magnetic field induced phase transition of CrSBr occurs.

Figure S18 shows the integrated PL intensities of X_B in pristine CrSBr and X^+ and X in WSe_2/CrSBr as a function of the magnetic field along the $\hat{c}$-axis. We observe that the PL intensity of the X_B of CrSBr continuously decreases up to the field-induced ferromagnetic transition ($B_{z\ sat} \approx 2.2$ T), remaining constant at higher magnetic fields. In contrast, the X and X^+ of WSe_2 exhibit a sudden increase in their PL intensities at around $B = 1.4$ T and $B = 1.8$ T, respectively. This behavior may indicate a possible resonant energy transfer between X_B in CrSBr and the X and X^+ in WSe_2 at selected magnetic fields, as such PL intensity improvement is not observed for X_B of CrSBr.

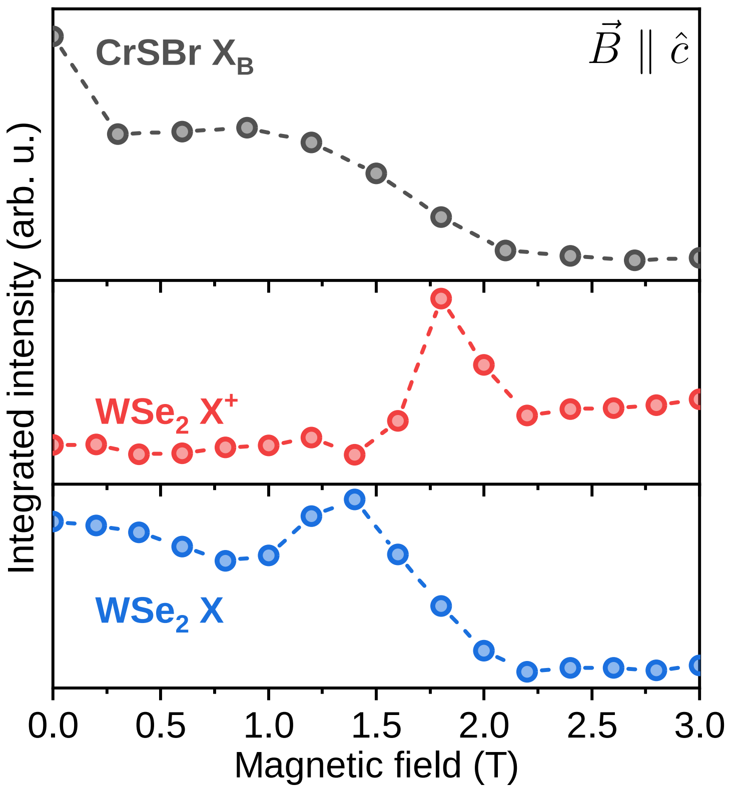

Figure S18: Comparison of the integrated circularly polarized PL intensities as a function of the magnetic field for the X_B of CrSBr, and the X and X^+ of WSe_2 in the WSe_2/CrSBr heterostructure. The PL measurements were performed using linearly polarized laser excitation along the $\hat{b}$-axis, with the magnetic field applied along the $\hat{c}$-axis at at 3.6 K .

Figure S19 illustrates the color-coded map of the linearly-polarized PL intensity of WSe_2/CrSBr heterostructure as a function of the angle of in-plane polarization and for different perpendicular magnetic field values ($\vec{B} \parallel \hat{c}$). We observed an enhancement in the PL intensity of the DDE PL peaks in WSe_2/CrSBr after the magnetic field-induced phase transition of CrSBr, when the PL polarization angle was measured along the in-plane $\hat{b}$-axis of CrSBr, suggesting an anisotropic RET mechanism.

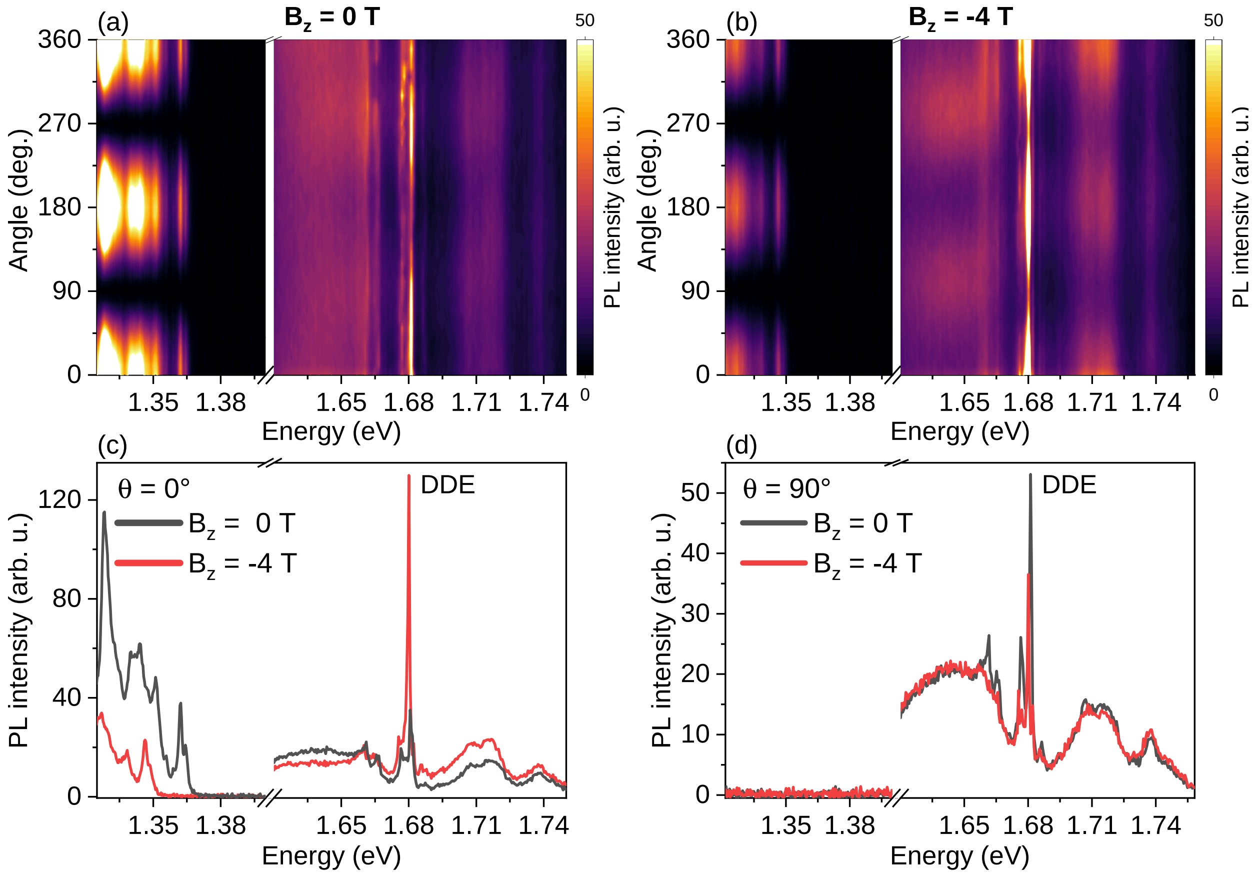

Figure S19: (a,b) Color-coded map of the linearly-polarized PL intensity as a function of the angle of in-plane polarization under 0 T and -4 T ($\vec{B} \parallel \hat{c}$), respectively. (c,d) PL spectra at 0 T and -4 T for in-plane polarization angles of 0° and 90°, respectively.

Figure S20 (a) shows the linearly polarized PL spectra for both WSe_2/CrSBr and the X_B of pristine CrSBr at 0.5 T ($\vec{B} \parallel \hat{b}$), for linearly polarization along the $\hat{b}$ and $\hat{a}$ axes. We observed that some DDE PL peaks of WSe_2/CrSBr exhibit stronger emission polarized along the $\hat{b}$ direction as compared to the $\hat{a}$ direction (hatched area in Figure S20 (a)). Additionally, as expected, the X_B in the pristine CrSBr also displays linearly polarized PL emission along the $\hat{b}$ axis (Figure S20 (b)). We remark that the observed enhanced PL intensity of WSe_2 cannot be attributed solely to the combined PL signals of the two materials. The relative increase in the PL emission of WSe_2/CrSBr at around 1.68 eV is approximately five times stronger as compared to the PL intensity of the X_B in pristine CrSBr. This result indicates that there is an additional mechanism that should be considered to explain the observed

increase of PL intensity of DDE in WSe_2/CrSBr after the magnetic phase transition of CrSBr.

Considering that the PL intensity of some emission peaks in WSe_2/CrSBr are reduced after the magnetic phase transition of CrSBr (notably the D, X^+, and X bands), the PL enhancement observed for the DDE peaks was associated with a RET between the X_B of CrSBr and DDE in WSe_2.

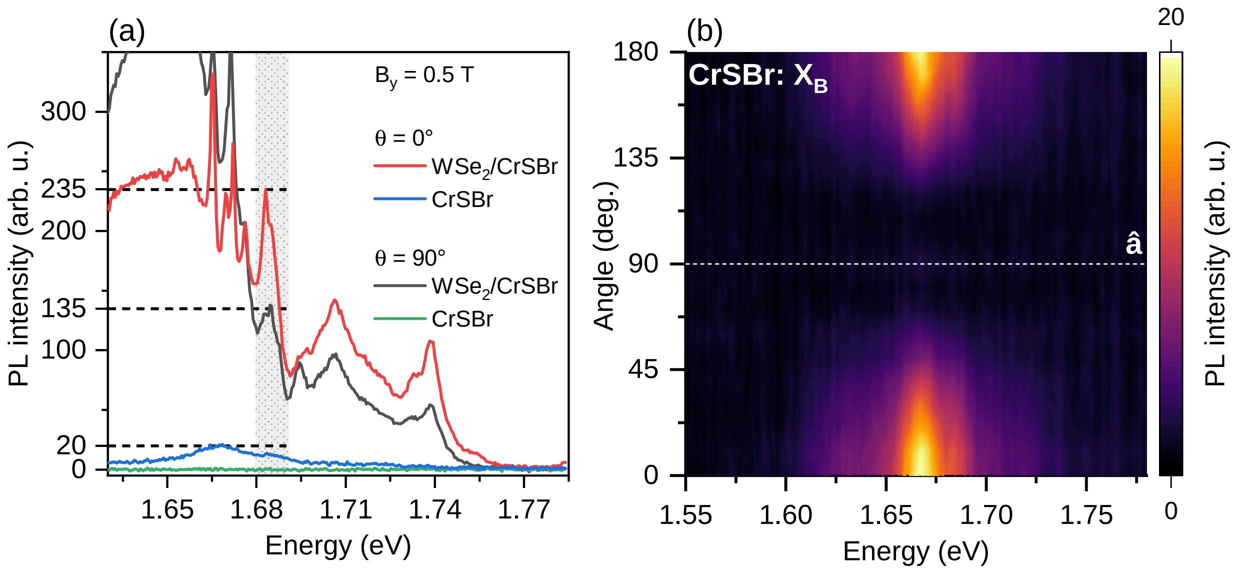

Figure S20: (a) PL spectra of pristine CrSBr and WSe_2/CrSBr for linearly polarization detection along the $\hat{b}$ ($\theta = 0°$) and $\hat{a}$ ($\theta = 90°$) crystallographic axes of CrSBr. (b) Color-coded map of the linearly polarized emission intensity of the X_B of CrSBr as a function of the in-plane polarization angle. All PL spectra were recorded at 3.6 K with linearly polarized excitation along the $\hat{b}$-axis.

Table 1 shows the PL intensity variation of DDE peaks at the resonant energy condition ($|B| = |B_{sat}|$), relative to the non-resonant condition ($|B| < |B_{sat}|$), for different sample positions. It was observed that only the DDE PL peaks with energies around the PL band of X_B centered at 1.687 eV (Figure S7(b)) exhibit PL intensity enhancement.

Table 1: PL intensity variation for the sharp emission peaks observed at different sample positions after the magnetic field induced FM order of CrSBr.

Peak energy (eV)	Intensity variation (%)	Sample position
1.6835	11.5	1
1.6815	22.9	
1.6748	0.0	
1.6694	-3.0	
1.6925	46.3	2
1.6851	21.1	
1.6799	19.3	
1.6737	-7.5	
1.6900	32.3	3
1.6821	28.4	
1.6770	-0.5	
1.6708	11.0	
1.6828	41.3	4
1.6821	12.3	
1.6804	4.1	
1.6778	-3.2	
1.6687	7.5	
1.6836	15.7	5
1.6810	23.0	
1.6768	48.8	
1.6727	-2.4	
1.6826	43.8	6
1.6821	12.3	
1.6778	3.3	
1.6683	-1.5	

Magneto-PL results for the sample hBN/WSe_2/hBN/CrSBr

In order to suppress the charge transfer effects and explore additional aspects of the RET in the WSe_2/CrSBr heterostructure, a similar heterostructure was prepared using a thin layer of hBN between the CrSBr and WSe_2 layers. Figure S21 presents the optical microscopy and AFM images for this hBN/WSe_2/hBN/CrSBr heterostructure. The hBN spacer layer between the CrSBr and WSe_2 layers has a thickness of (2.4 ± 0.4) nm, while the CrSBr layer is (19 ± 2) nm thick.

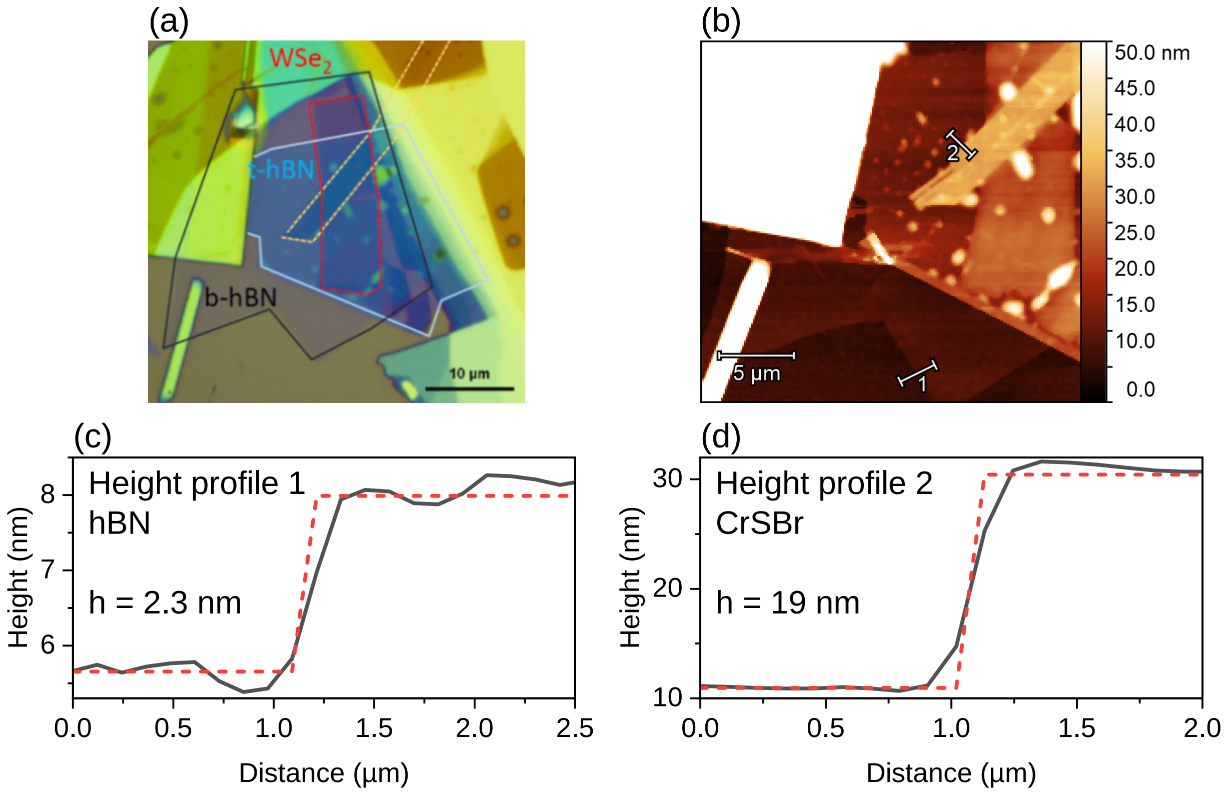

Figure S21: (a) Optical microscopy and (b) AFM images of the hBN/WSe_2/hBN/CrSBr heterostructure. (c) Height profile of the hBN spacer layer and (d) CrSBr flake, showing height variations along direction 1 and 2 in panel (b). t-hBN and b-hBN refer to the hBN layers on top of the sample and between the WSe_2 and CrSBr layers, respectively.

Circularly polarized magneto-PL experiments for this sample were also performed under a perpendicular magnetic field up to 3 T ($\vec{B} \parallel \hat{c}$). These magneto-PL results are presented in Figure S22.

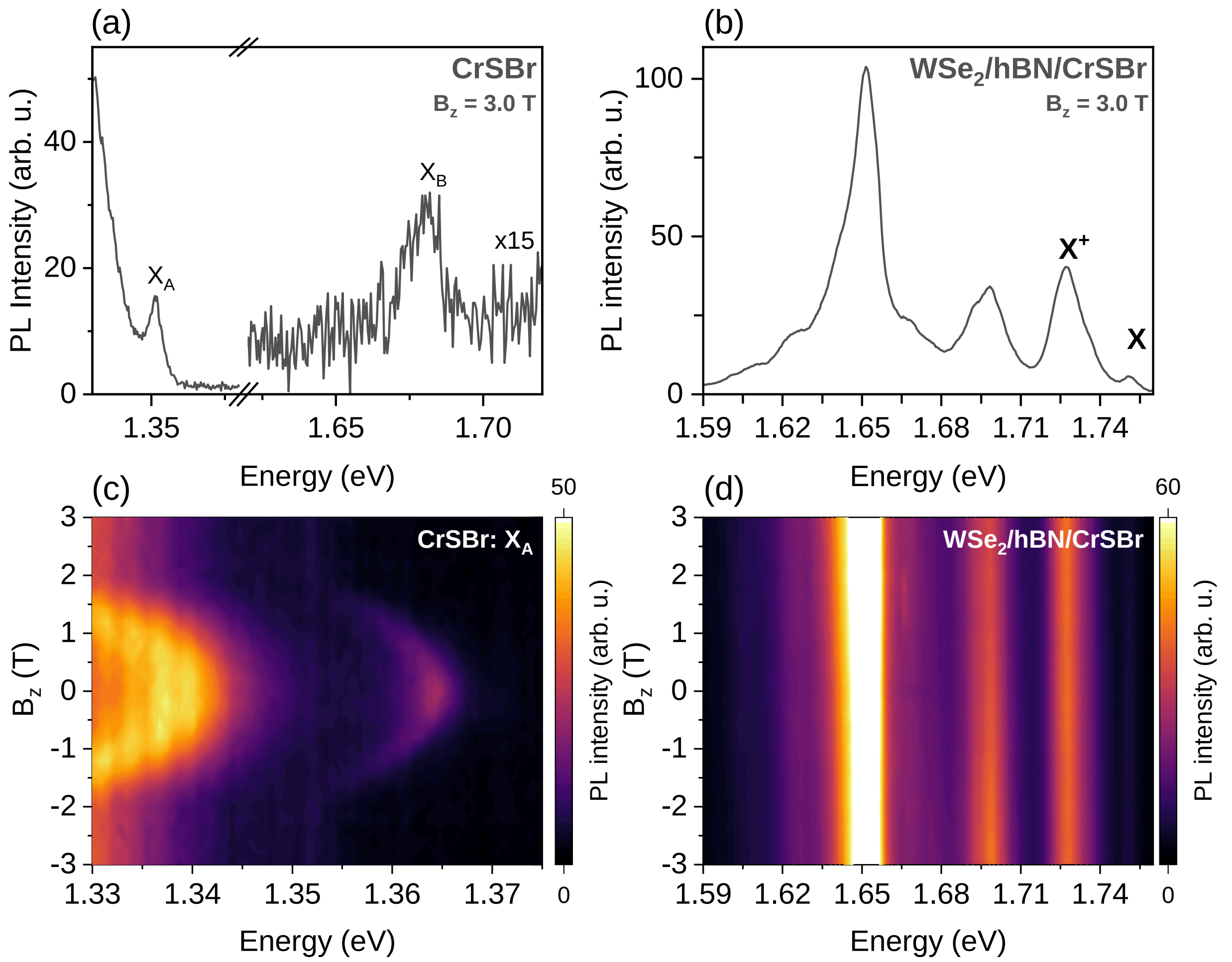

Figure S22: (a,b) PL spectra for CrSBr and WSe_2 in the WSe_2/CrSBr heterostructure respectively, in the hBN/WSe_2/hBN/CrSBr heterostructure at $B_z = 3$ T. (c,d) Color-coded maps of the PL intensity of CrSBr and WSe_2 respectively as a function of the magnetic field applied along the $\hat{c}$-axis. The PL spectra were measured at 3.6 K using linearly polarized excitation along the $\hat{b}$-axis with a laser energy of 1.88 eV and right circularly polarized (σ^+) PL detection for positive magnetic fields.

We observe that the magnetic field dependence of the X_A of CrSBr has a saturation field of about 2 T which is similar to previous results in the literature (Figure S22 (c)). Furthermore, no important changes were observed for the magnetic field dependence of the PL intensity of WSe_2 as compared to the WSe_2/CrSBr sample (Figure S22 (d)). The observed changes in the magnetic field dependence of the PL intensity and the PL peak energy in hBN/WSe_2/hBN/CrSBr sample can be solely explained considering the valley Zeeman effect.

The effective suppression of energy transfer effects in the PL spectra in the WSe_2/hBN/CrSBr heterostructure, demonstrates that the RET in the WSe_2/CrSBr heterostructure occurs over very short distances. This result suggests that the RET occurs in a very near-field interaction, and is probably dependent on the overlap of the electron wave functions in ML-TMD and CrSBr, and therefore is consistent with a Dexter-type mechanism. However, further studies are required to fully elucidate the nature of RET in this heterostructure.

Time resolved PL measurements

Figure S23(a) shows the TRPL results at 3.6 K for the trion in monolayer WSe_2 on WSe_2/CrSBr and WSe_2/SiO_2 samples at 0 T, whereas Figure S23(b) presents the evolution of the trion decay profile in the WSe_2/CrSBr sample under 0 T and 1.8 T (resonant condition) out-plane magnetic fields.

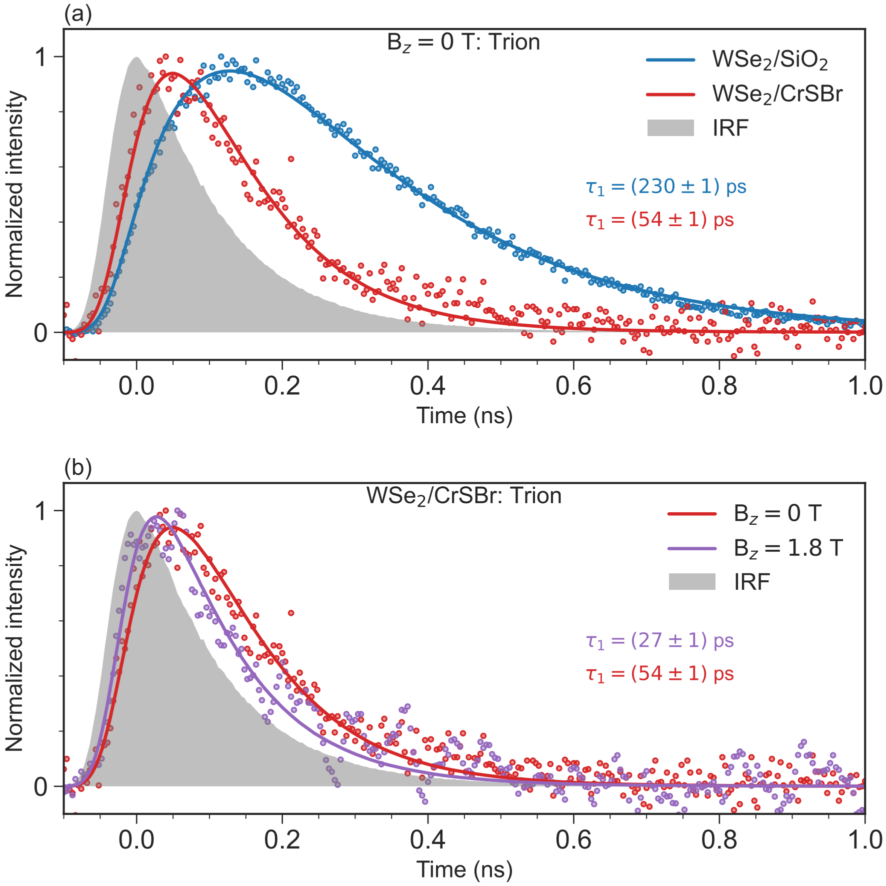

Figure S23: Time-resolved PL results. (a) Faster decay times are observed for the trion in the WSe_2/CrSBr sample as compared to the WSe_2/SiO_2 reference at $B_z = 0$ T. (b) Trion decay times in WSe_2/CrSBr heterostructure under different applied magnetic fields. The gray filled curve represents the instrument response function (IRF) of the experimental setup. The solid lines are convoluted fittings of exponential decays with the IRF.

The decay times were extracted by fitting Equation S3 to experimental data, which considers the convolution of exponential decays with the instrument response function (IRF):

$$y(t) = \int_0^t \mathrm{IRF}(t') \cdot \sum_i A_i e^{-(t-t')/\tau_i}\, dt' \tag{S3}$$

where A_i is the amplitude of the decay component and τ_i is the corresponding decay constant.

The trion decay in the WSe_2/SiO_2 reference is satisfactorily described by a single exponential decay with $\tau_1 = (230\pm1)$ ps. The PL decay curve in the WSe_2/CrSBr heterostructure was also fitted using a single exponential model. However, a reduction in the trion lifetime

($\tau_1 = (54 \pm 1)$ ps) in the WSe_2/CrSBr heterostructure at 0 T is observed (Figure S23(a)). The observed reduction of time decay is in agreement with previously reported fast exciton dynamics induced by charge transfer processes in vdW heterostructures[25–27].

We have also measured TRPL under out of plane magnetic field. We observed that at under resonant energy condition between the trion in WSe_2 and X_B in CrSBr (for a out of plane magnetic field of 1.8 T), the PL lifetime of the trion became even shorter. A time constant of $\tau_1 = (27 \pm 1)$ ps, which is on the order of the response of our system, was extracted by fitting a single exponential model to the experimental data.

References

(1) Serati de Brito, C.; Faria Junior, P. E.; Ghiasi, T. S.; Ingla-Aynés, J.; Rabahi, C. R.; Cavalini, C.; Dirnberger, F.; Mañas-Valero, S.; Watanabe, K.; Taniguchi, T.; Zollner, K.; Fabian, J.; Schüller, C.; van der Zant, H. S. J.; Gobato, Y. G. Charge transfer and asymmetric coupling of MoSe2 valleys to the magnetic order of CrSBr. *Nano Letters* **2023**, *23*, 11073–11081.

(2) Beer, A.; Zollner, K.; Serati de Brito, C.; Faria Junior, P. E.; Parzefall, P.; Ghiasi, T. S.; Ingla-Aynés, J.; Mañas-Valero, S.; Boix-Constant, C.; Watanabe, K.; others Proximity-Induced Exchange Interaction and Prolonged Valley Lifetime in MoSe2/CrSBr Van-Der-Waals Heterostructure with Orthogonal Spin Textures. *ACS Nano* **2024**, *18*, 31044–31054.

(3) Kang, J.; Tongay, S.; Zhou, J.; Li, J.; Wu, J. Band offsets and heterostructures of two-dimensional semiconductors. *Applied Physics Letters* **2013**, *102*.

(4) Faria Junior, P. E.; Fabian, J. Signatures of electric field and layer separation effects on the spin-valley physics of MoSe2/WSe2 Heterobilayers: from energy bands to dipolar Excitons. *Nanomaterials* **2023**, *13*, 1187.

(5) Linhart, L.; Paur, M.; Smejkal, V.; Burgdörfer, J.; Mueller, T.; Libisch, F. Localized intervalley defect excitons as single-photon emitters in WSe 2. *Physical Review Letters* **2019**, *123*, 146401.

(6) Cavalini, C.; Rabahi, C.; de Brito, C. S.; Lee, E.; Toledo, J. R.; Cazetta, F. F.; Fernandes de Oliveira, R. B.; Andrade, M. B.; Henini, M.; Zhang, Y.; Kim, J.; Barcelos, I. D.; Galvão Gobato, Y. Revealing localized excitons in WSe2/β-Ga2O3. *Applied Physics Letters* **2024**, *124*.

(7) de Brito, C. S.; Rabahi, C. R.; Teodoro, M. D.; Franco, D. F.; Nalin, M.; Barcelos, I. D.; Gobato, Y. G. Strain engineering of quantum confinement in WSe2 on nano-roughness glass substrates. *Applied Physics Letters* **2022**, *121*.

(8) Serati de Brito, C.; Rosa, B. L.; Chaves, A.; Cavalini, C.; Rabahi, C. R.; Franco, D. F.; Nalin, M.; Barcelos, I. D.; Reitzenstein, S.; Gobato, Y. G. Probing the nature of single-photon emitters in a WSe2 monolayer by magneto-photoluminescence spectroscopy. *Nano Letters* **2024**, *24*, 13300–13306.

(9) Datta, B. et al. Magnon-mediated exciton–exciton interaction in a van der Waals antiferromagnet. *Nature Materials* **2025**, *24*, 1027–1033.

(10) Komar, R.; Łopion, A.; Goryca, M.; Rybak, M.; Woźniak, T.; Mosina, K.; Söll, A.; Sofer, Z.; Pacuski, W.; Faugeras, C.; Birowska, M.; Kossacki, P.; Kazimierczuk, T. Colossal magneto-excitonic effects in 2d van der Waals magnetic semiconductor CrSBr. *arXiv (cond-mat.mtrl-sci)* **Aug 30, 2024**, DOI: 10.48550/arXiv.2409.00187 (accessed 2025-08-19).

(11) Shi, J.; Wang, D.; Jiang, N.; Xin, Z.; Zheng, H.; Shen, C.; Zhang, X.; Liu, X. Giant Magneto-Exciton Coupling in 2D van der Waals CrSBr. *ACS Nano* **0**, *0*, null, PMID: 40802066.

(12) Nessi, L.; Occhialini, C. A.; Demir, A. K.; Powalla, L.; Comin, R. Magnetic Field Tunable Polaritons in the Ultrastrong Coupling Regime in CrSBr. *ACS Nano* **2024**, *18*, 34235–34243, PMID: 39639608.

(13) Robert, C.; Amand, T.; Cadiz, F.; Lagarde, D.; Courtade, E.; Manca, M.; Taniguchi, T.; Watanabe, K.; Urbaszek, B.; Marie, X. Fine structure and lifetime of dark excitons in transition metal dichalcogenide monolayers. *Physical Review B* **2017**, *96*, 155423.

(14) Srivastava, A.; Sidler, M.; Allain, A. V.; Lembke, D. S.; Kis, A.; Imamoğlu, A. Optically active quantum dots in monolayer WSe2. *Nature Nanotechnology* **2015**, *10*, 491–496.

(15) He, Y.-M.; Clark, G.; Schaibley, J. R.; He, Y.; Chen, M.-C.; Wei, Y.-J.; Ding, X.; Zhang, Q.; Yao, W.; Xu, X.; Lu, C.-Y.; Pan, J.-W. Single quantum emitters in monolayer semiconductors. *Nature Nanotechnology* **2015**, *10*, 497–502.

(16) Chakraborty, C.; Kinnischtzke, L.; Goodfellow, K. M.; Beams, R.; Vamivakas, A. N. Voltage-controlled quantum light from an atomically thin semiconductor. *Nature Nanotechnology* **2015**, *10*, 507–511.

(17) Ren, S.; Tan, Q.; Zhang, J. Review on the quantum emitters in two-dimensional materials. *Journal of Semiconductors* **2019**, *40*, 071903.

(18) Kumar, S.; Kaczmarczyk, A.; Gerardot, B. D. Strain-induced spatial and spectral isolation of quantum emitters in mono-and bilayer WSe2. *Nano Letters* **2015**, *15*, 7567–7573.

(19) Blundo, E.; Polimeni, A. Alice (and Bob) in Flatland. *Nano Letters* **2024**, *24*, 9777–9783.

(20) Woźniak, T.; Faria Junior, P. E.; Seifert, G.; Chaves, A.; Kunstmann, J. Exciton g factors of van der Waals heterostructures from first-principles calculations. *Physical Review B* **2020**, *101*, 235408.

(21) Koperski, M.; Molas, M. R.; Arora, A.; Nogajewski, K.; Slobodeniuk, A. O.; Faugeras, C.; Potemski, M. Optical properties of atomically thin transition metal dichalcogenides: observations and puzzles. *Nanophotonics* **2017**, *6*, 1289–1308.

(22) Koperski, M.; Molas, M. R.; Arora, A.; Nogajewski, K.; Bartos, M.; Wyzula, J.; Vaclavkova, D.; Kossacki, P.; Potemski, M. Orbital, spin and valley contributions to Zeeman splitting of excitonic resonances in MoSe2, WSe2 and WS2 Monolayers. *2D Materials* **2018**, *6*, 015001.

(23) Förste, J.; Tepliakov, N. V.; Kruchinin, S. Y.; Lindlau, J.; Funk, V.; Förg, M.; Watanabe, K.; Taniguchi, T.; Baimuratov, A. S.; Högele, A. Exciton g-factors in monolayer and bilayer WSe2 from experiment and theory. *Nature Communications* **2020**, *11*, 4539.

(24) Li, Z.; Wang, T.; Lu, Z.; Jin, C.; Chen, Y.; Meng, Y.; Lian, Z.; Taniguchi, T.; Watanabe, K.; Zhang, S.; Smirnov, D.; Shi, S.-F. Revealing the biexciton and trion-exciton complexes in BN encapsulated WSe2. *Nature Communications* **2018**, *9*, 3719.

(25) Bai, Z.; Zhang, H.; He, J.; He, D.; Wang, J.; Wu, W.; Zhang, Y.; Wang, W.; Wang, Y.; Yu, X.; others Ultrafast Decay of Interlayer Exciton in WS2/MoSe2 Heterostructure Under Pressure. *Advanced Electronic Materials* **2025**, *11*, 2400333.

(26) He, J.; Wang, C.; Zhou, B.; Zhao, Y.; Tao, L.; Zhang, H. 2D van der Waals heterostructures: processing, optical properties and applications in ultrafast photonics. *Materials Horizons* **2020**, *7*, 2903–2921.

(27) Hong, X.; Kim, J.; Shi, S.-F.; Zhang, Y.; Jin, C.; Sun, Y.; Tongay, S.; Wu, J.; Zhang, Y.; Wang, F. Ultrafast charge transfer in atomically thin MoS2/WS2 heterostructures. *Nature nanotechnology* **2014**, *9*, 682–686.
